# Supplementary figures and images for: Binding of LncDACH1 to dystrophin impairs the membrane trafficking of Nav1.5 protein and increases ventricular arrhythmia susceptibility
Source: eLife. 2025 Jan 7;12:RP89690. doi: 10.7554/eLife.89690 (PMC11706603; doi:10.7554/eLife.89690)

**Figure1 A**

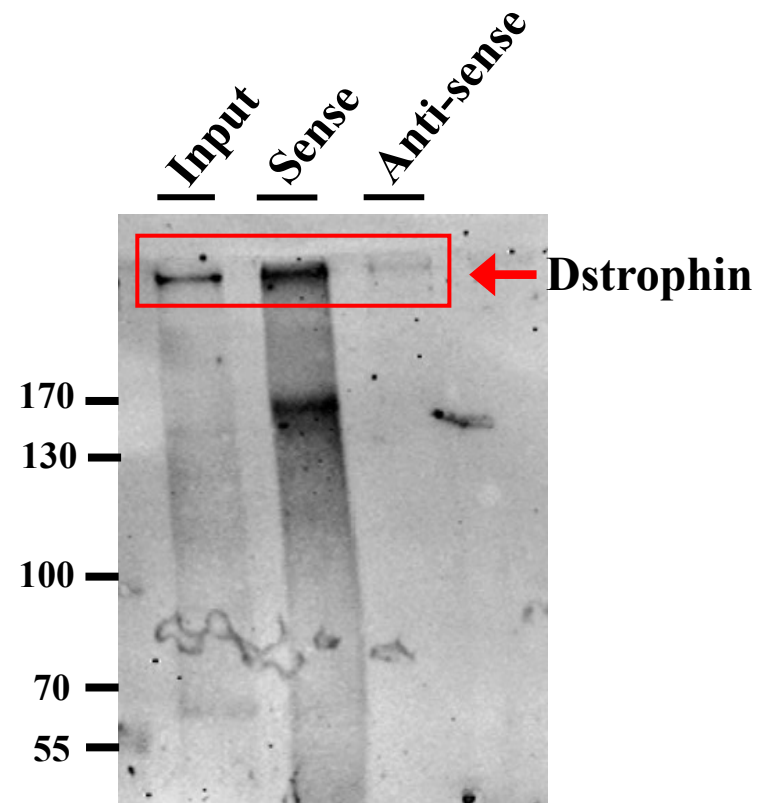

Supplement: Figure 1—source data 2. [file elife-89690-fig1-data2.pdf]

Figure1

C

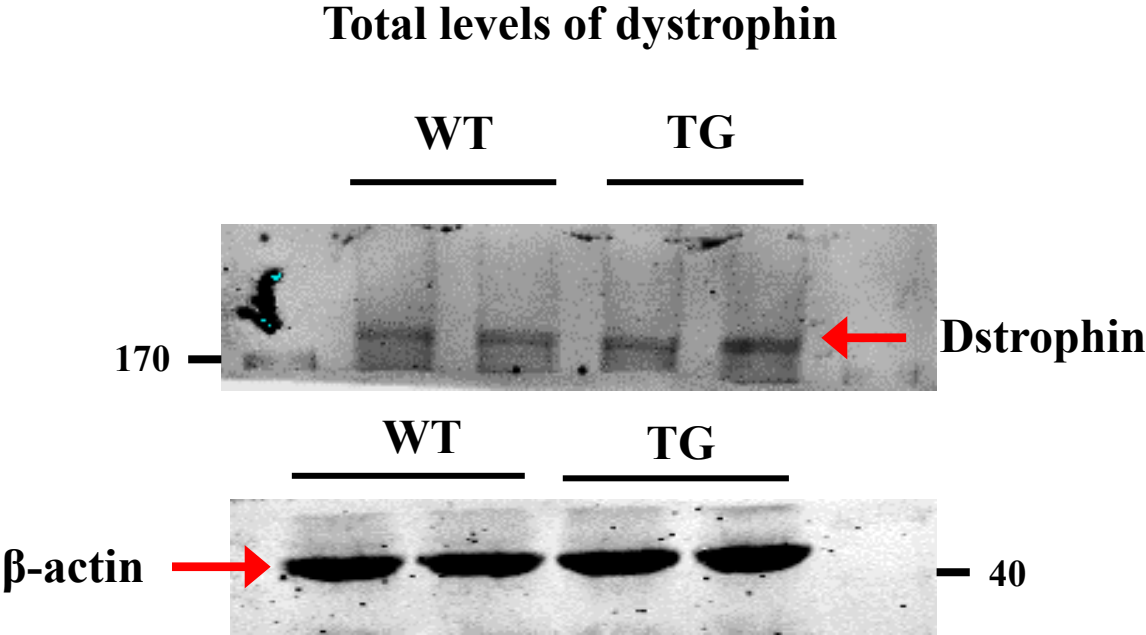

Figure1

C

Membrane levels of dystrophin

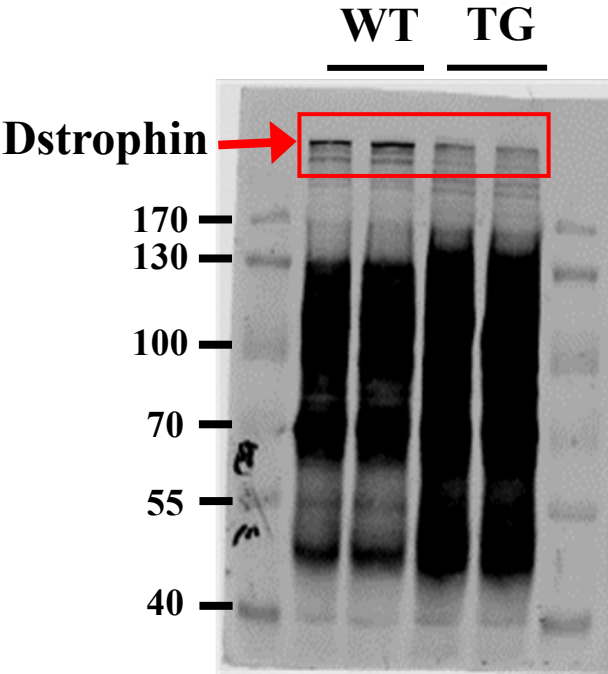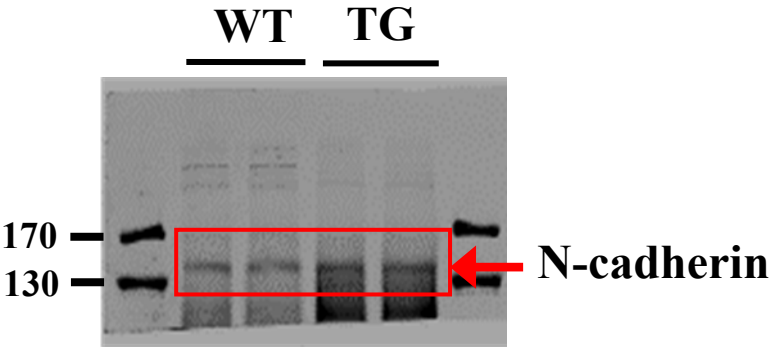

Figure1

C

Intracellular levels of dystrophin

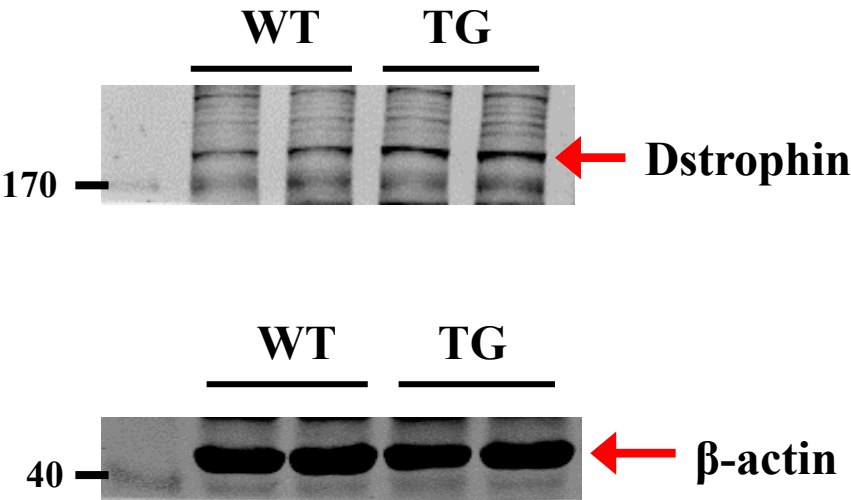

Supplement: Figure 1—source data 4. [file elife-89690-fig1-data4.pdf]

Figure1

D

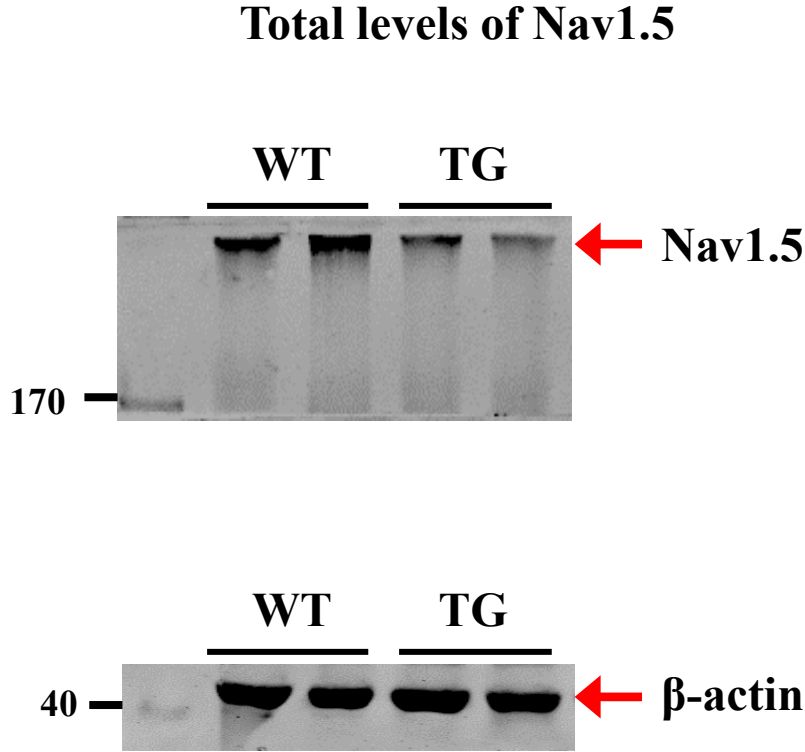

**Figure1**

**D**

**Membrane levels of Nav1.5**

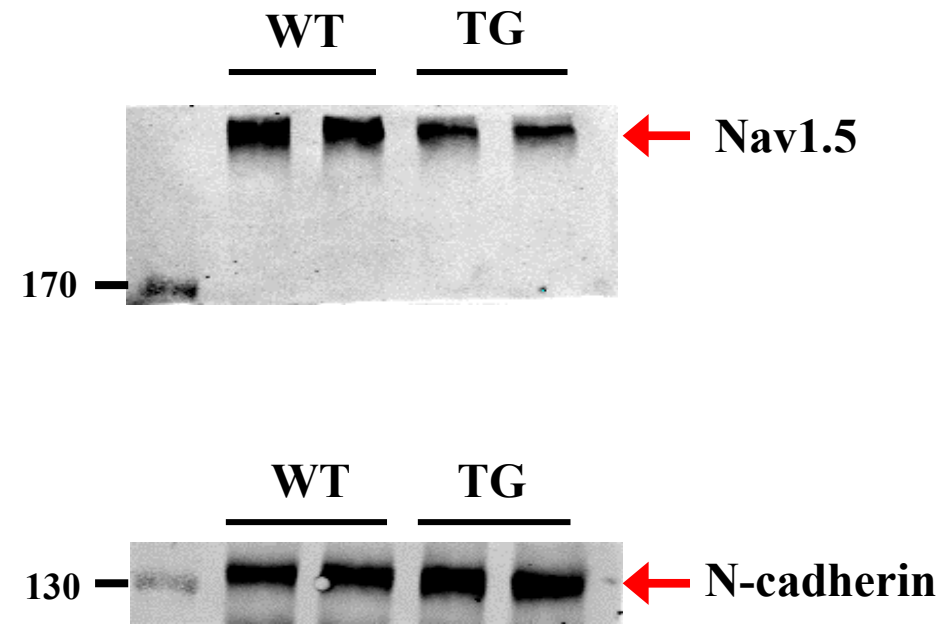

Figure1

D

Intracellular levels of Nav1.5

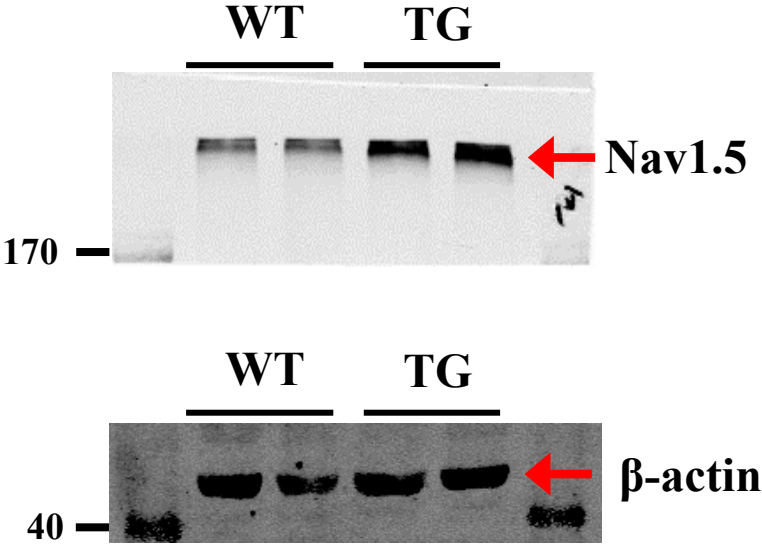

Supplement: Figure 1—source data 6. [file elife-89690-fig1-data6.pdf]

**Fig 1-figure supplement 1A**

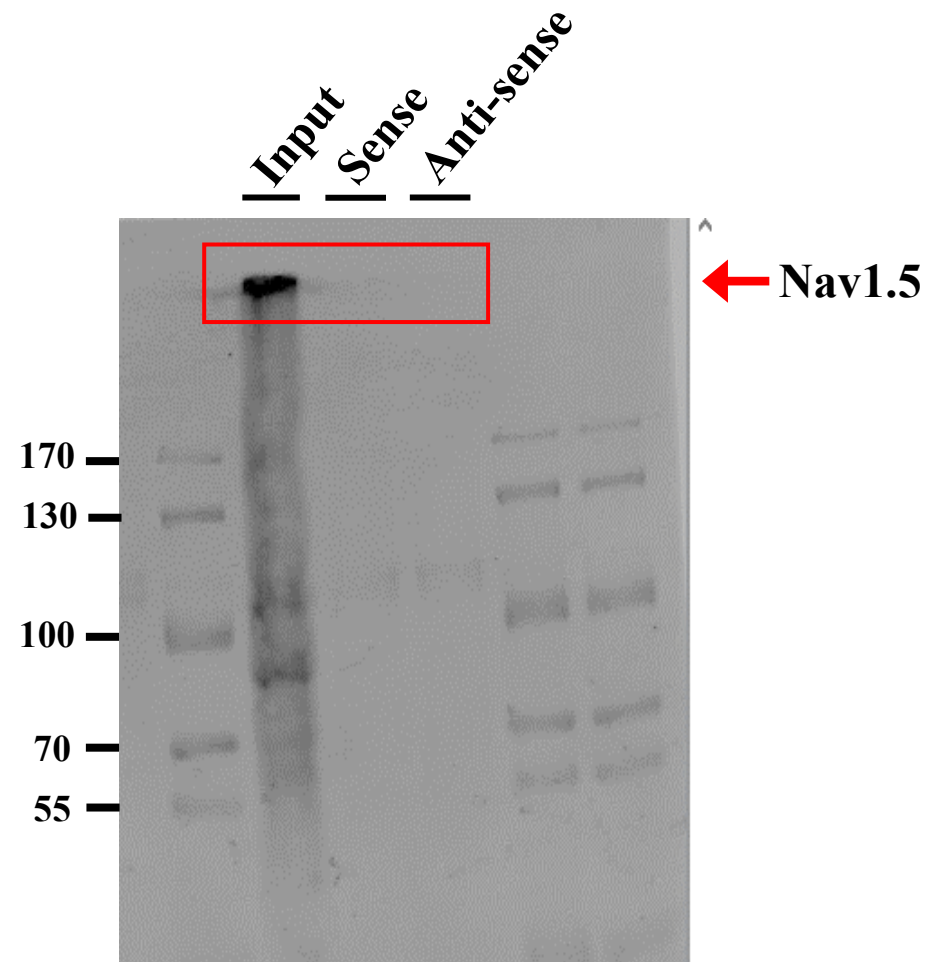

Supplement: Figure 1—figure supplement 1—source data 2. [file elife-89690-fig1-figsupp1-data2.pdf]

Figure3

B

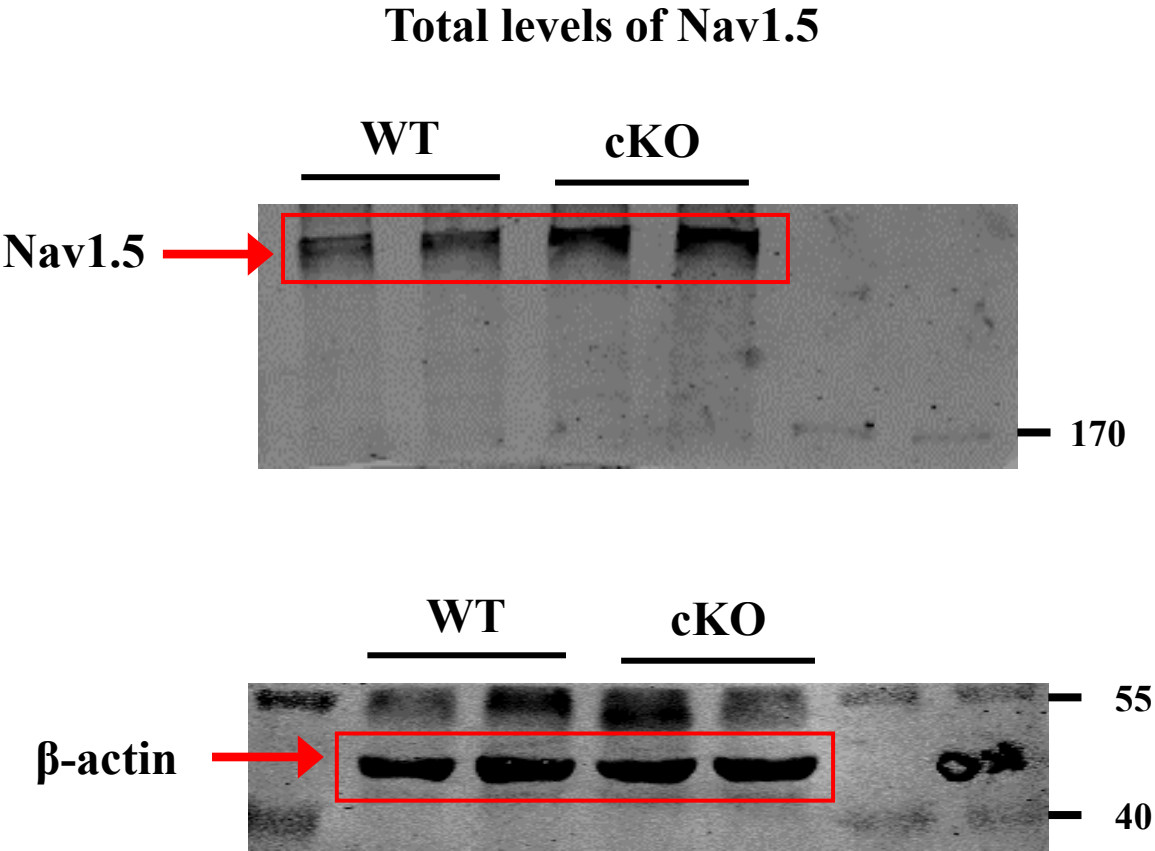

**Figure3**

**B**

**Membrane levels of Nav1.5**

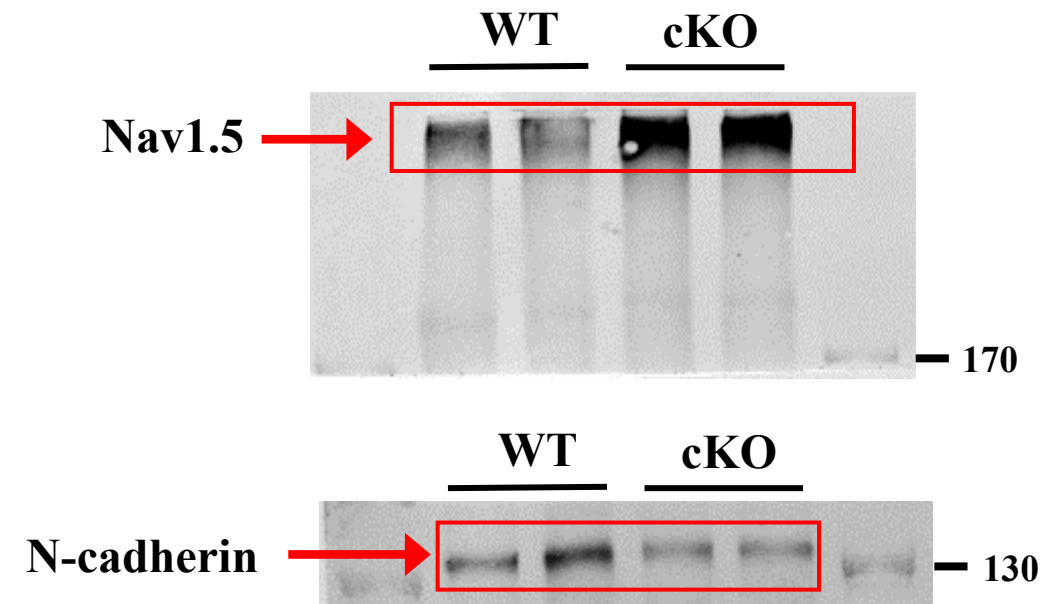

**Figure3**

**B**

**Intracellular levels of Nav1.5**

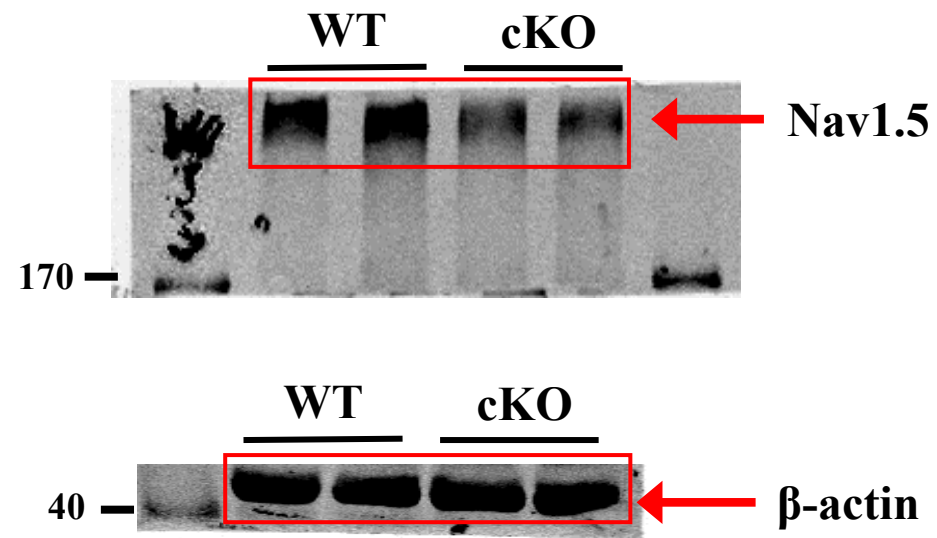

Supplement: Figure 3—source data 4. [file elife-89690-fig3-data4.pdf]

Figure6

A

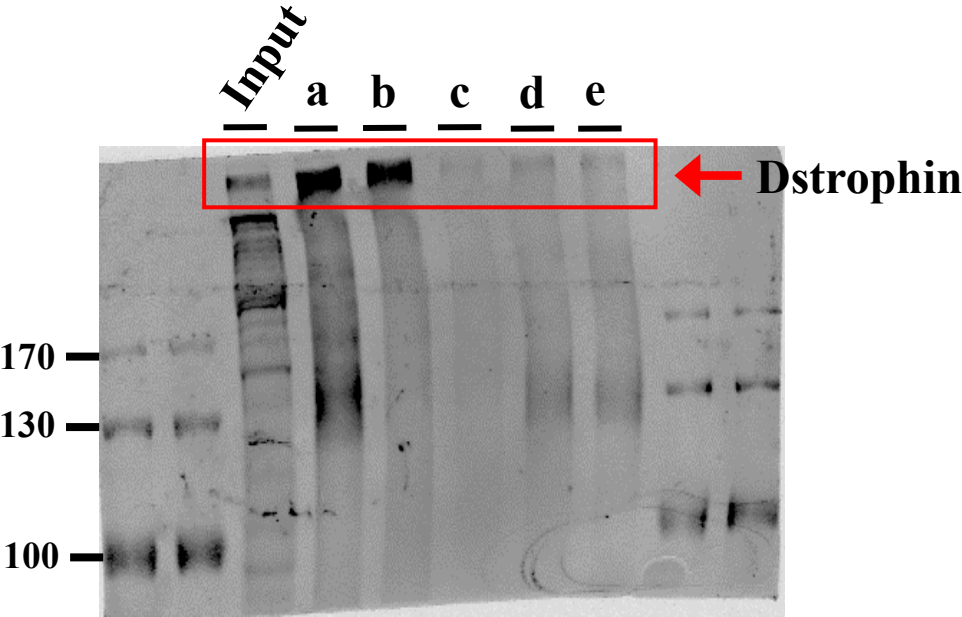

Supplement: Figure 6—source data 2. [file elife-89690-fig6-data2.pdf]

Figure7

A

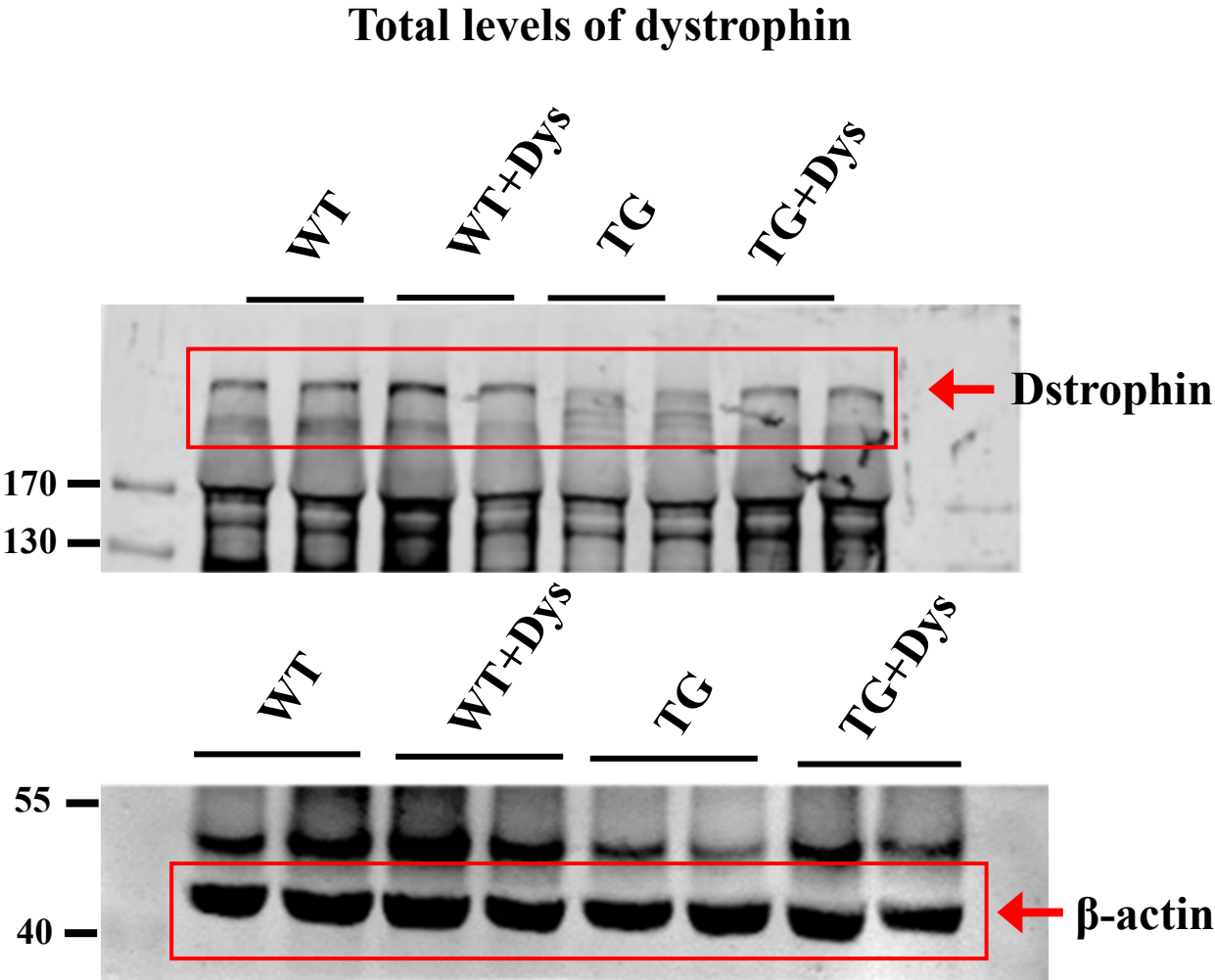

Figure7

A

Membrane levels of dystrophin

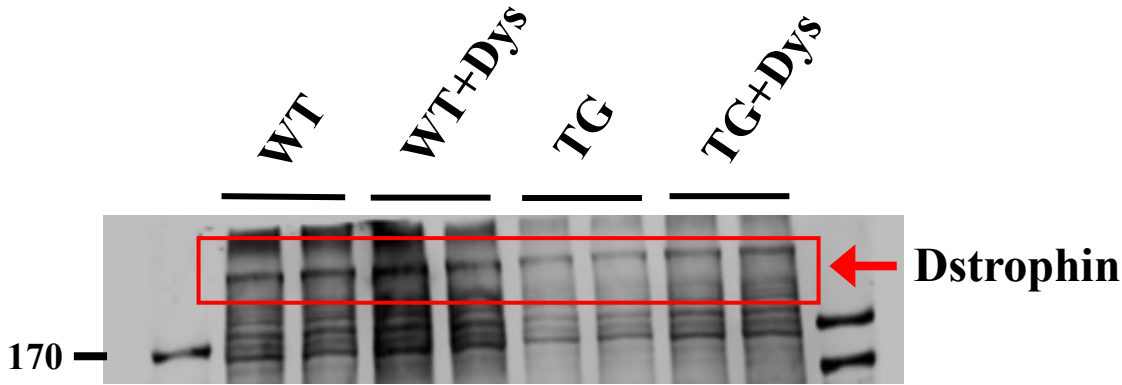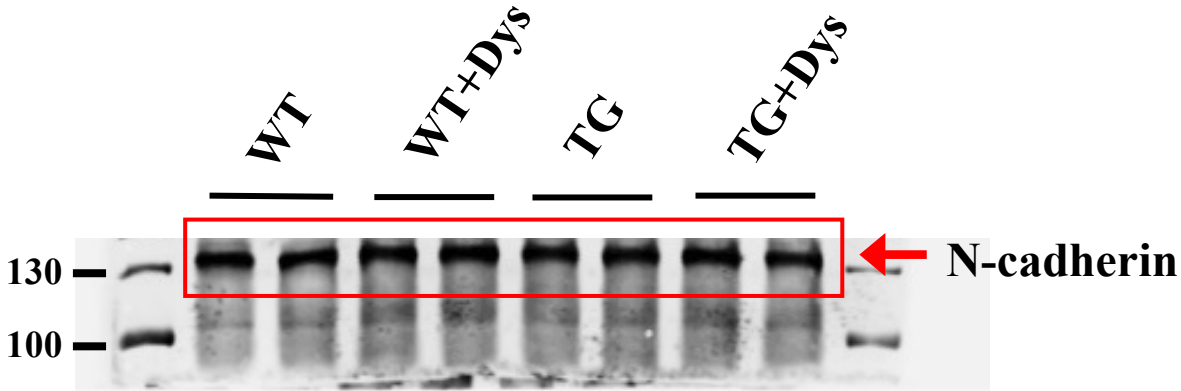

Figure7

A

Intracellular levels of dystrophin

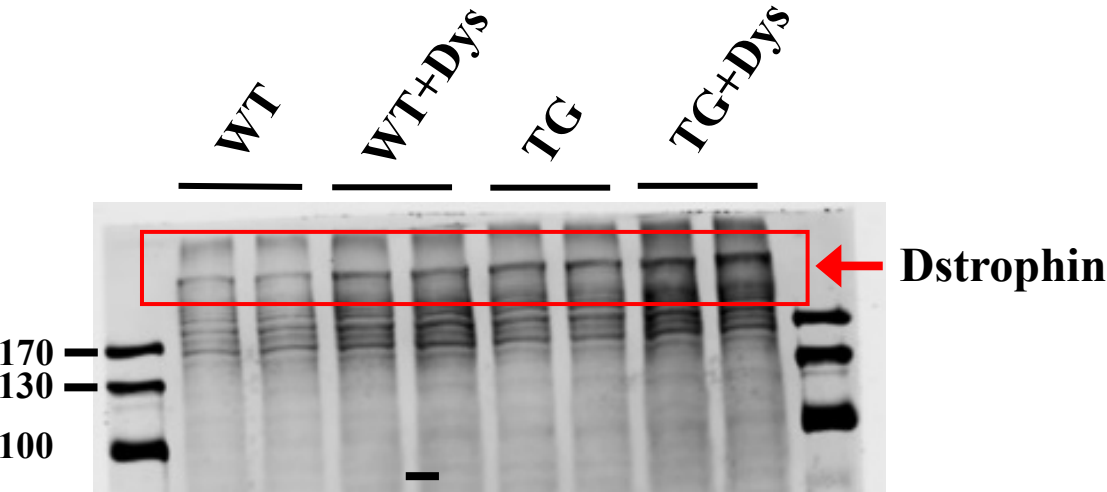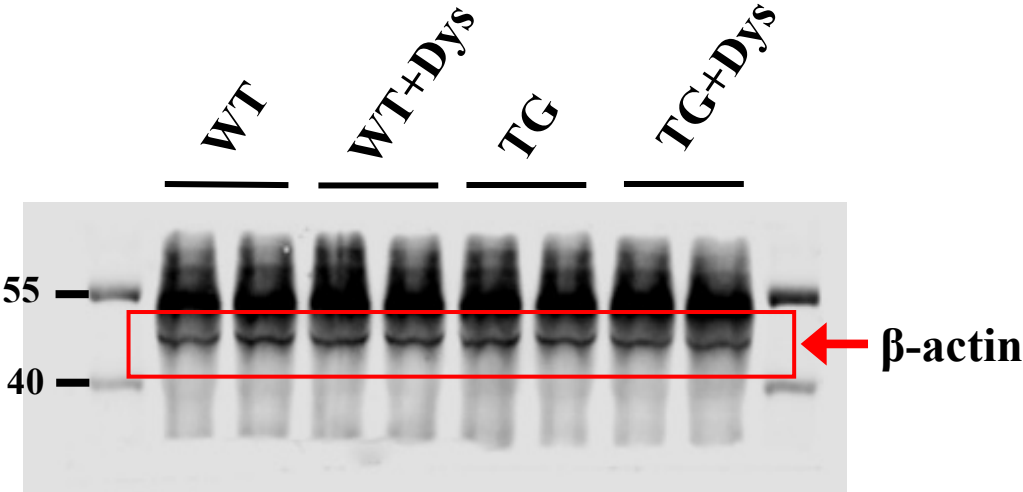

Supplement: Figure 7—source data 2. [file elife-89690-fig7-data2.pdf]

Figure 8

E

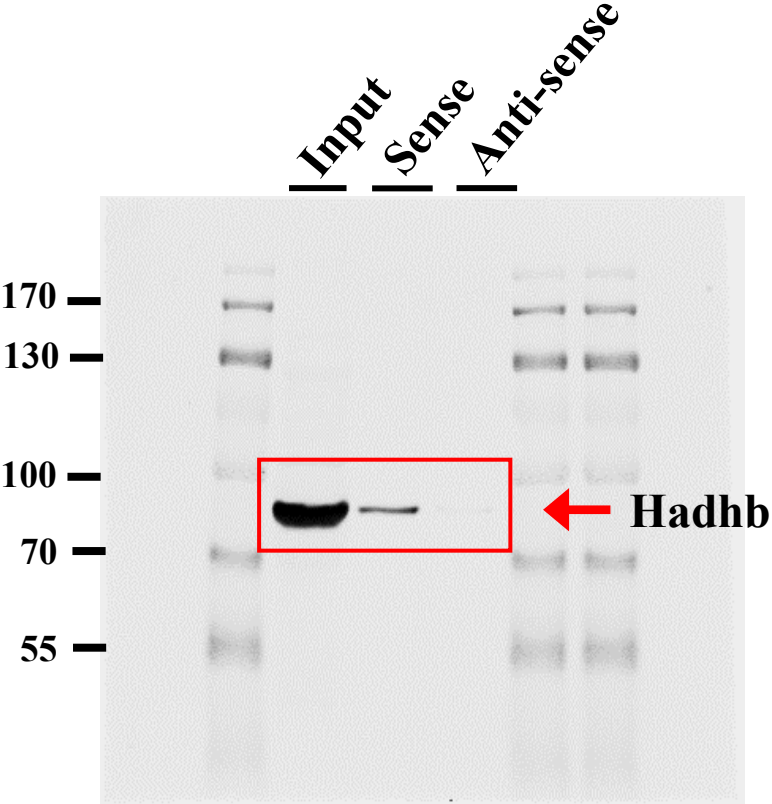

Supplement: Figure 8—source data 2. [file elife-89690-fig8-data2.pdf]

**Figure 8**

**F**

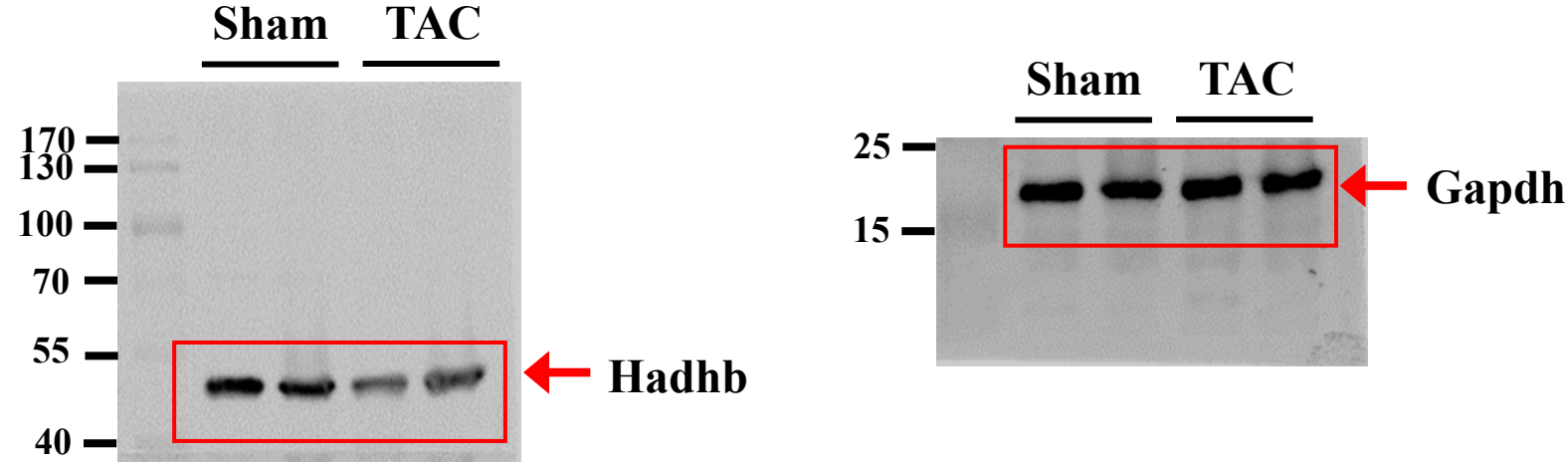

Supplement: Figure 8—source data 4. [file elife-89690-fig8-data4.pdf]

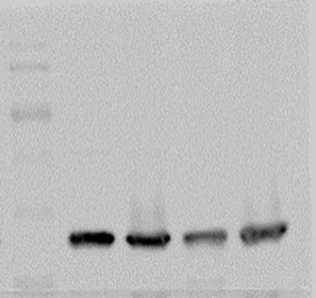

Supplement: Figure 8—source data 5. [file elife-89690-fig8-data5.zip › Fig8-data5(Hadhb).png]

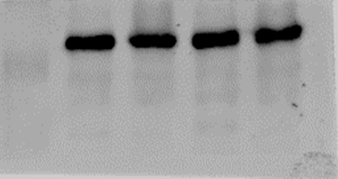

Supplement: Figure 8—source data 5. [file elife-89690-fig8-data5.zip › Fig8-data5(Gapdh).png]

Figure 8

G

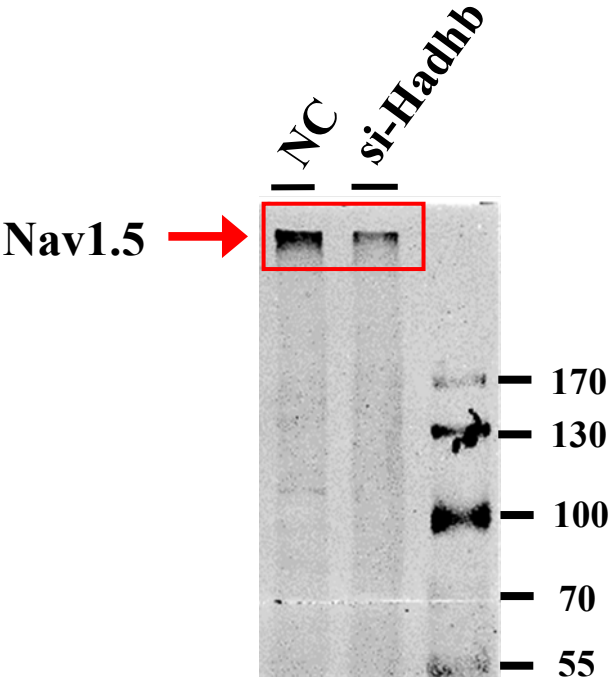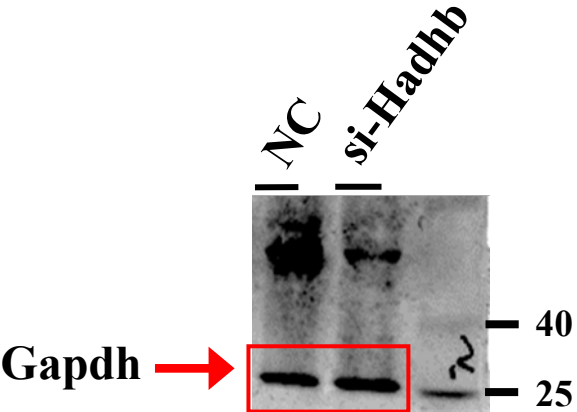

Supplement: Figure 8—source data 6. [file elife-89690-fig8-data6.pdf]

Figure 8-figure supplement 3

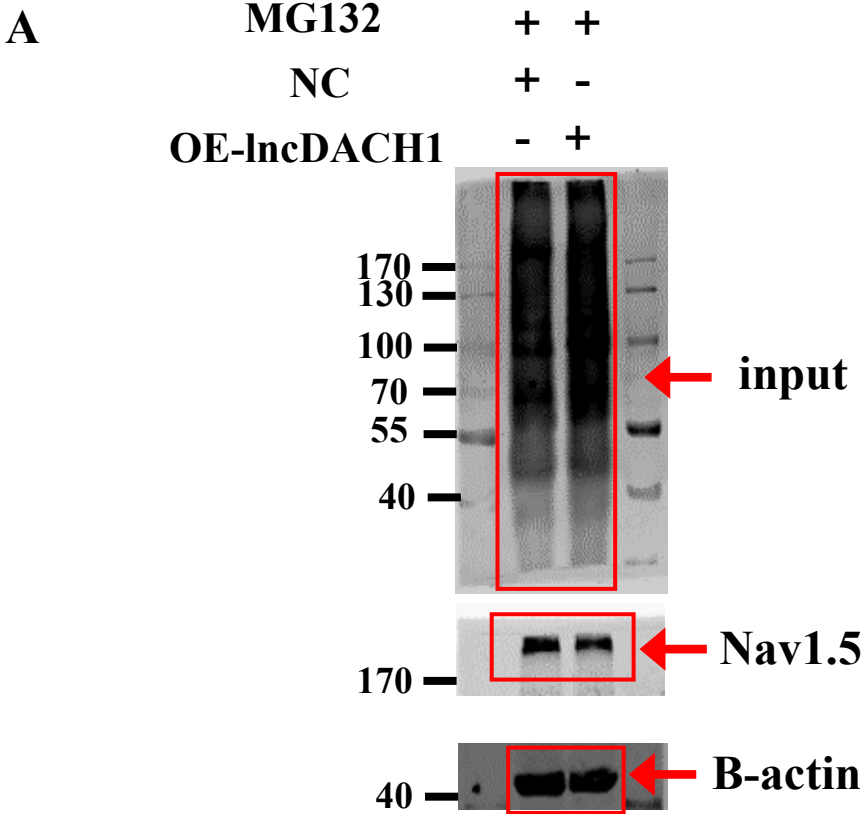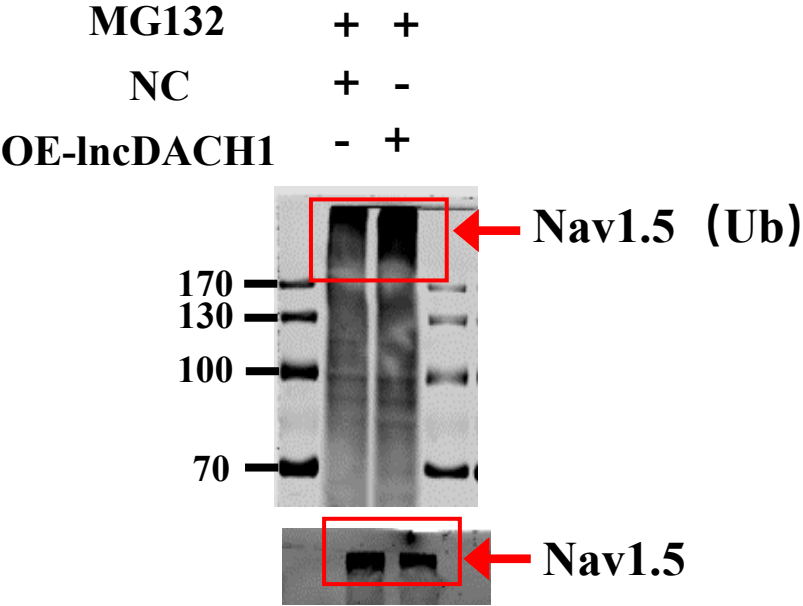

Supplement: Figure 8—figure supplement 3—source data 1. [file elife-89690-fig8-figsupp3-data1.pdf]

Figure 8-figure supplement 3

B

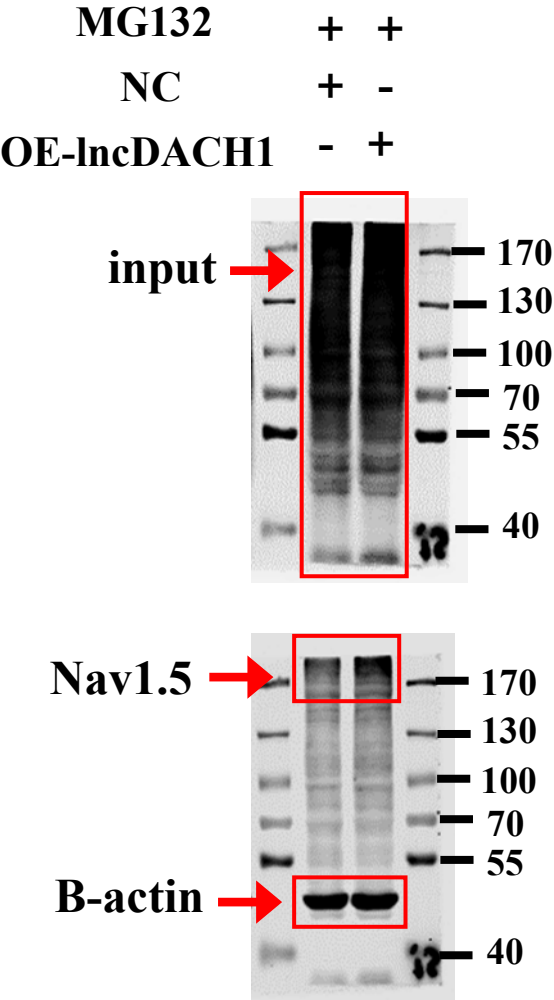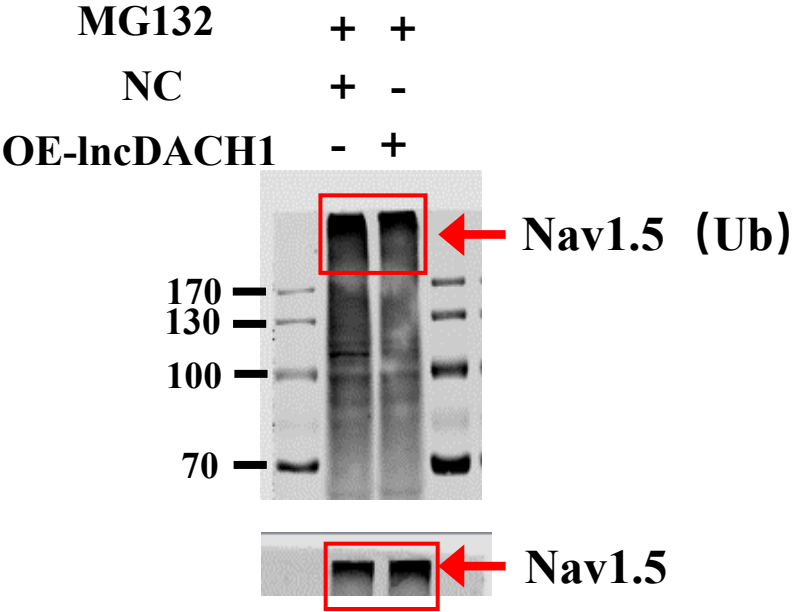

Supplement: Figure 8—figure supplement 3—source data 3. [file elife-89690-fig8-figsupp3-data3.pdf]
