# Supplementary material for: Binding of LncDACH1 to dystrophin impairs the membrane trafficking of Nav1.5 protein and increases ventricular arrhythmia susceptibility
Source: eLife. 2025 Jan 7;12:RP89690. doi: 10.7554/eLife.89690 (PMC11706603; doi:10.7554/eLife.89690)

Figure 2-figure supplement 1

A

Total levels of dystrophin

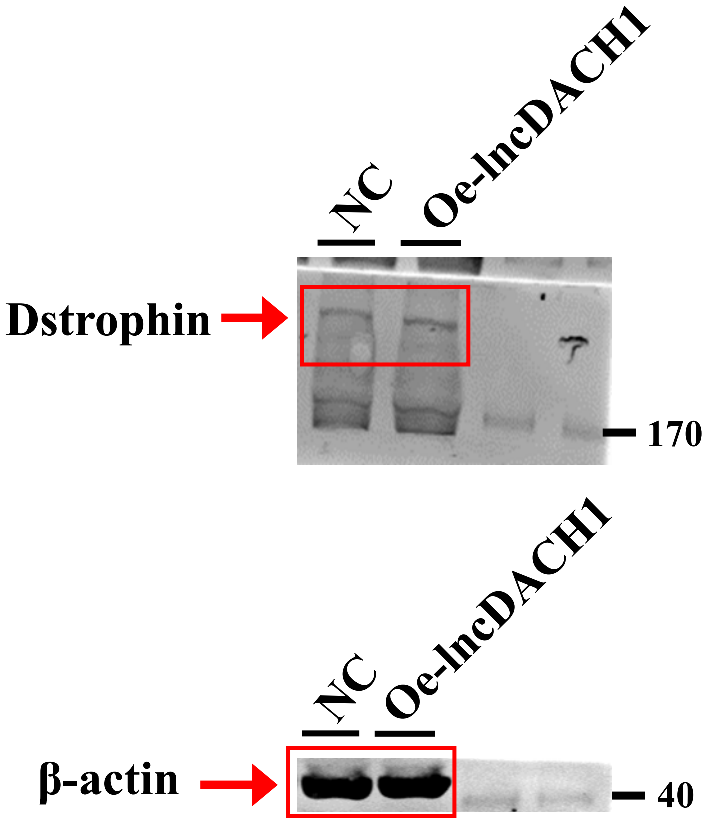

Figure 2-figure supplement 1

A

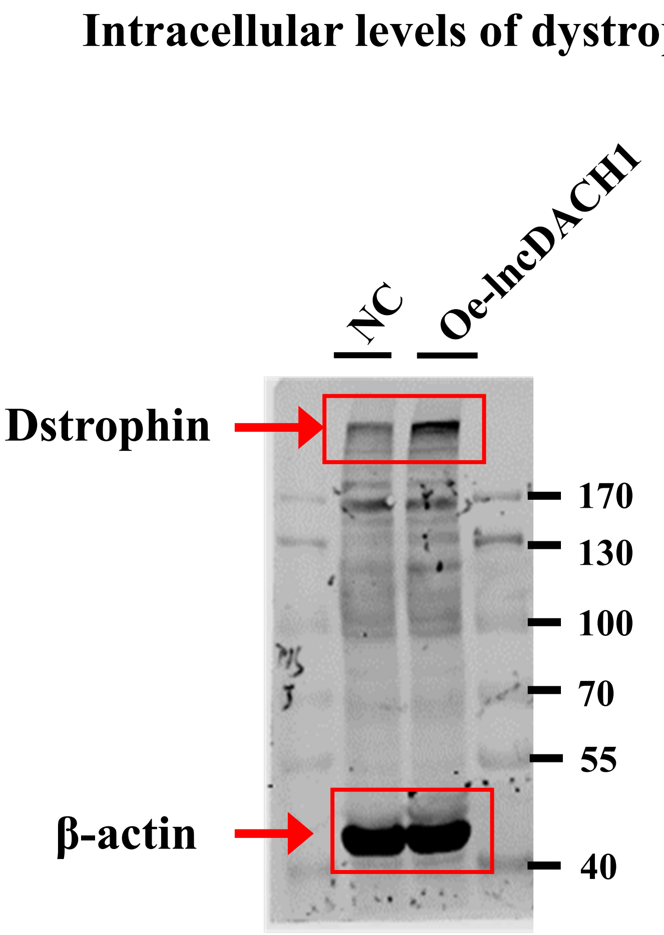

Figure 2-figure supplement 1

A

Membrane levels of dystrophin

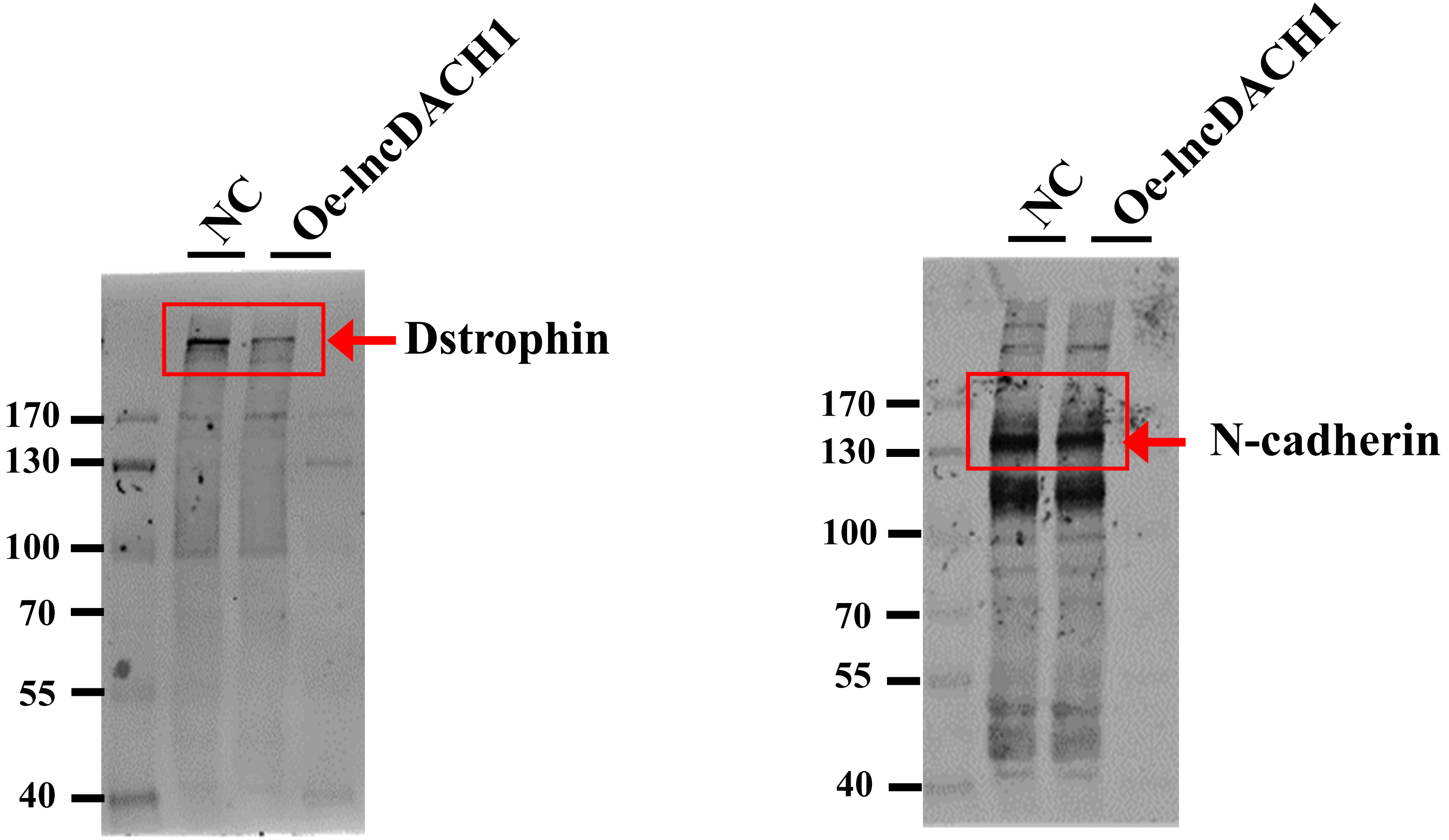

Supplement: Figure 2—figure supplement 1—source data 2. [file elife-89690-fig2-figsupp1-data2.pdf]
