# Supplementary material for: Binding of LncDACH1 to dystrophin impairs the membrane trafficking of Nav1.5 protein and increases ventricular arrhythmia susceptibility
Source: eLife. 2025 Jan 7;12:RP89690. doi: 10.7554/eLife.89690 (PMC11706603; doi:10.7554/eLife.89690)
Supplement: Figure 3—source data 2. [file elife-89690-fig3-data2.pdf]

Figure3

A

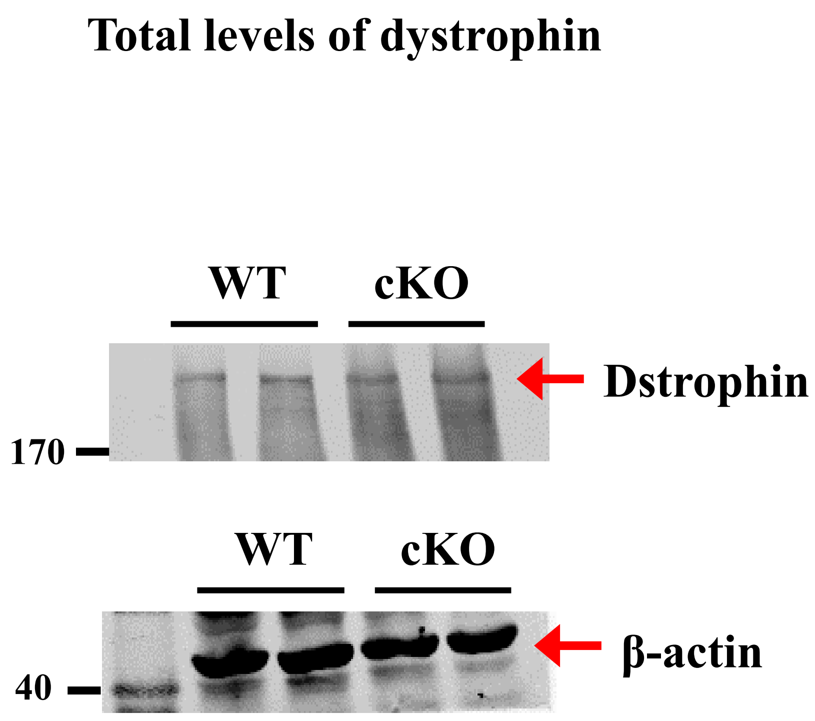

Total levels of dystrophin
WT
cKO
Dstrophin
170
WT
cKO
β-actin
40

Figure3

A

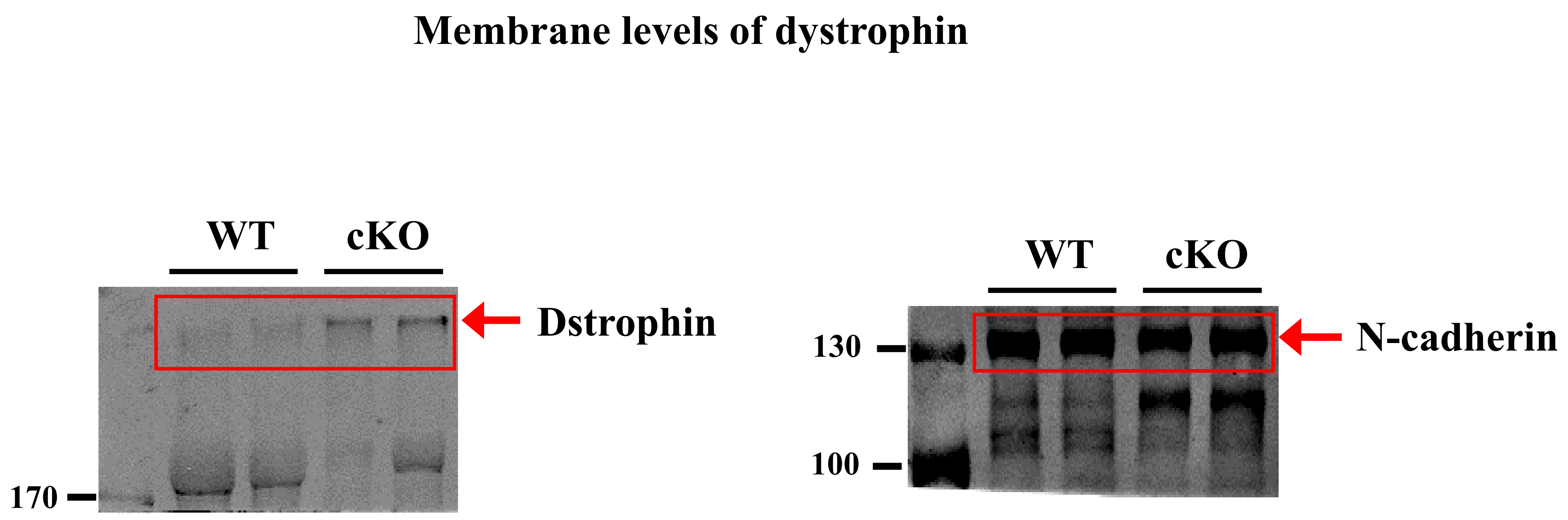

Membrane levels of dystrophin
WT
cKO
Dstrophin
170
WT
cKO
N-cadherin
130
100

Figure3

A

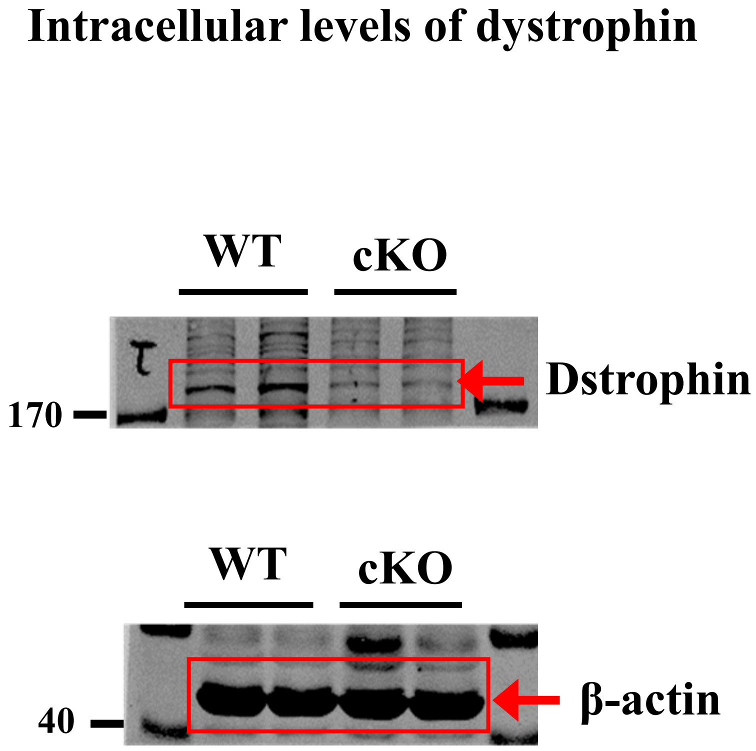
Intracellular levels of dystrophin
WT
cKO
170
Dstrophin
WT
cKO
40
β-actin
