# Supplementary material for: Binding of LncDACH1 to dystrophin impairs the membrane trafficking of Nav1.5 protein and increases ventricular arrhythmia susceptibility
Source: eLife. 2025 Jan 7;12:RP89690. doi: 10.7554/eLife.89690 (PMC11706603; doi:10.7554/eLife.89690)

Figure 4-figure supplement 1

B

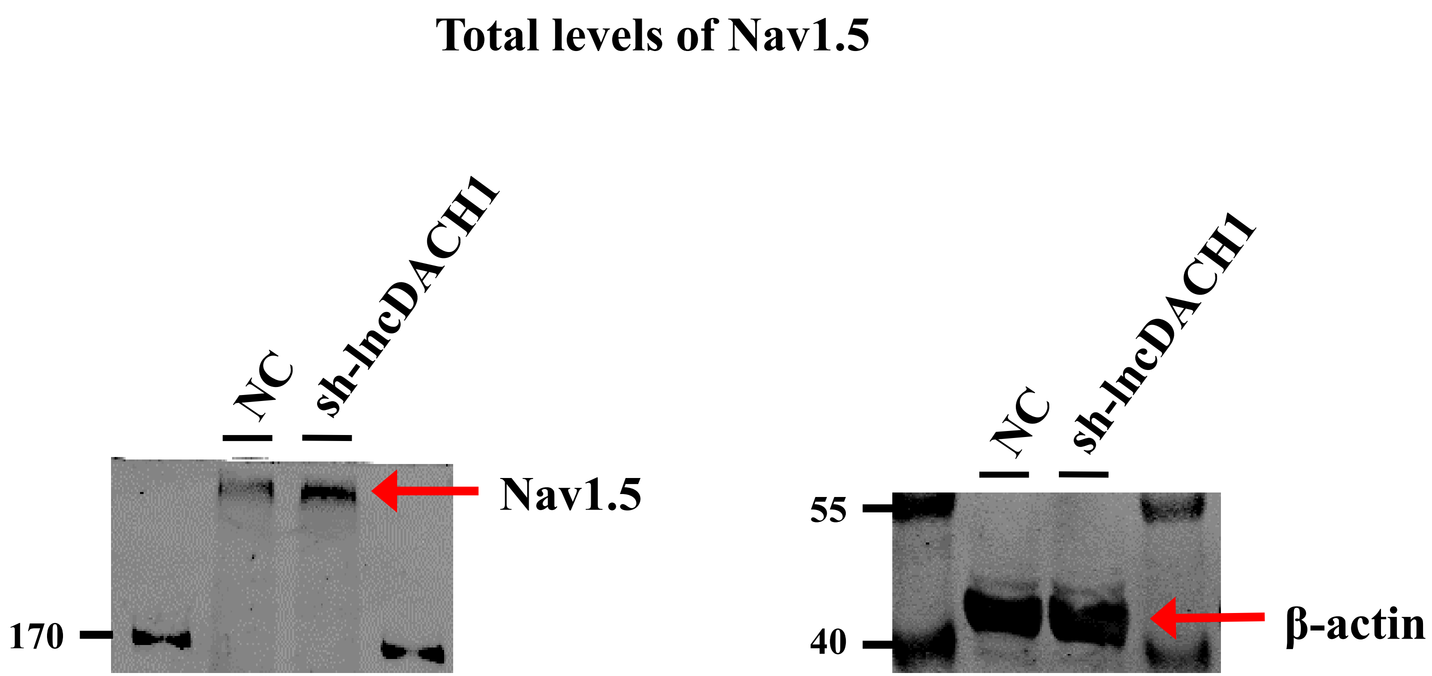

Figure 4-figure supplement 1

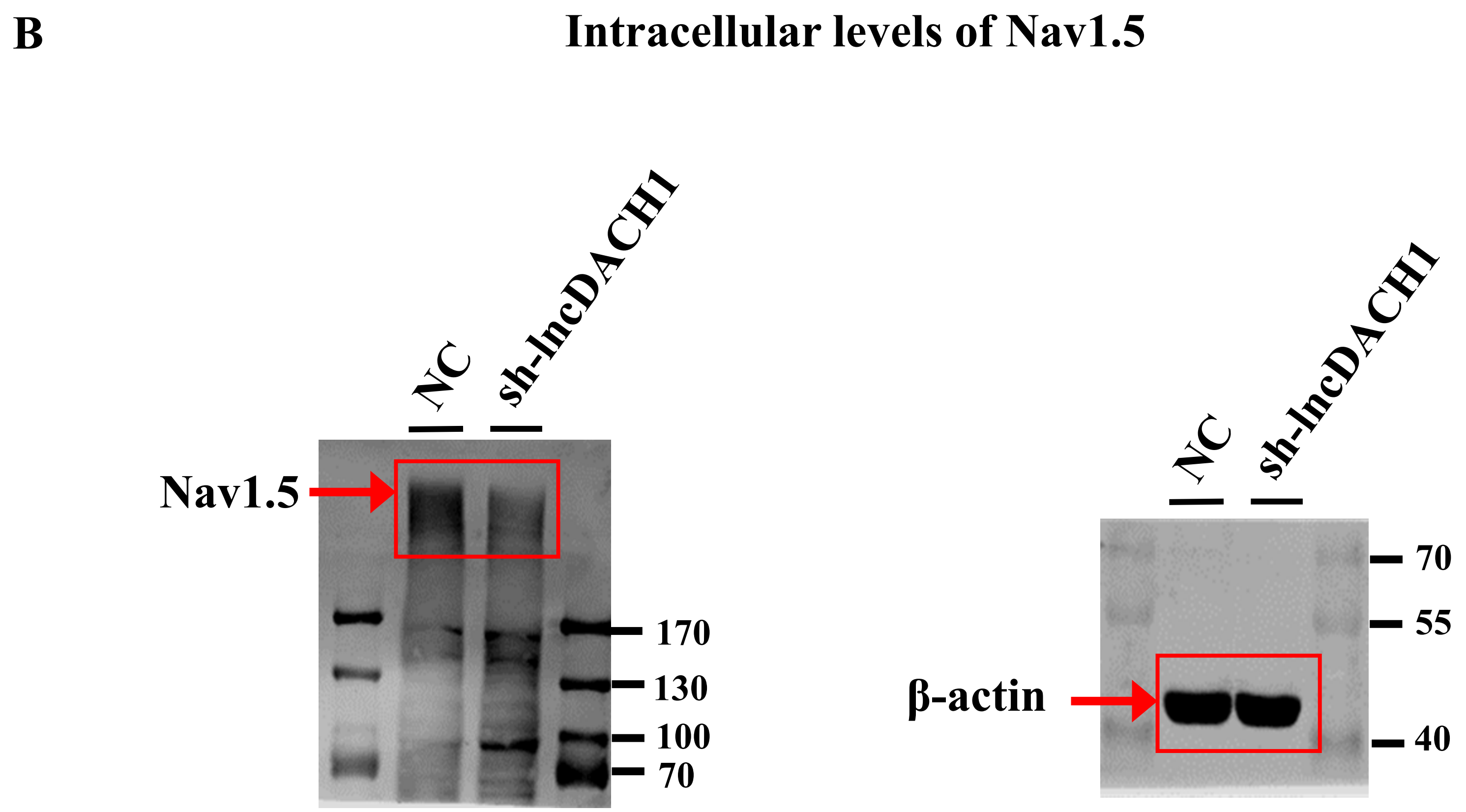

Figure 4-figure supplement 1

B

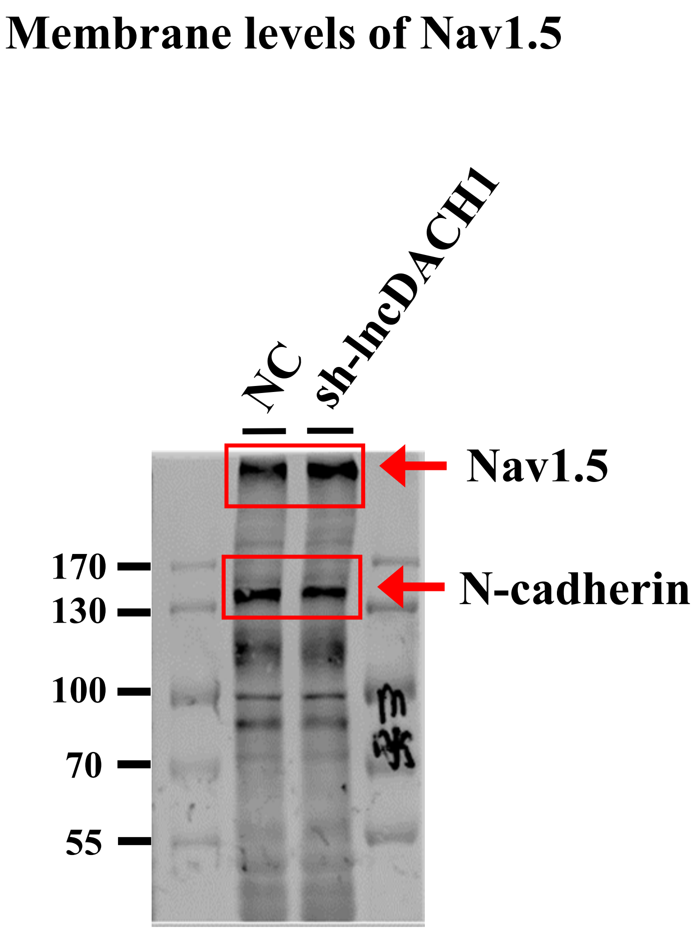

Supplement: Figure 4—figure supplement 1—source data 4. [file elife-89690-fig4-figsupp1-data4.pdf]
