# Supplementary material for: Binding of LncDACH1 to dystrophin impairs the membrane trafficking of Nav1.5 protein and increases ventricular arrhythmia susceptibility
Source: eLife. 2025 Jan 7;12:RP89690. doi: 10.7554/eLife.89690 (PMC11706603; doi:10.7554/eLife.89690)

Figure6

C

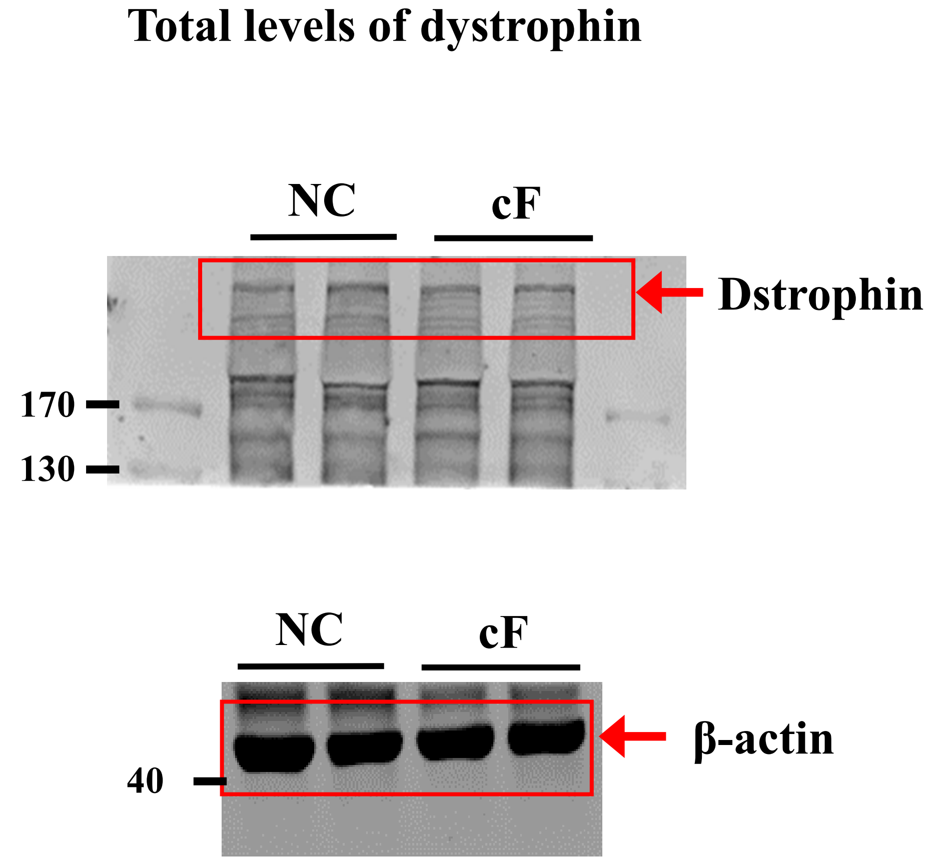

Total levels of dystrophin
NC
cF
Dstrophin
170
130
NC
cF
β-actin
40

Figure6

C

Membrane levels of dystrophin

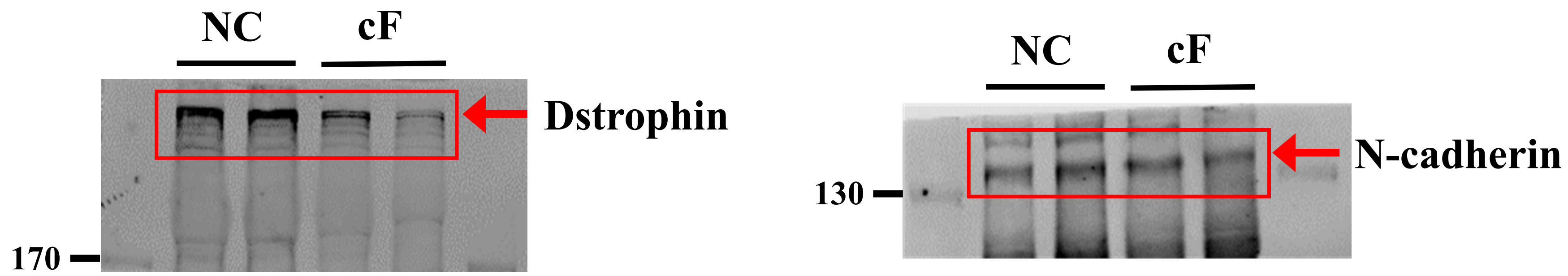

Figure6

C

Intracellular levels of dystrophin

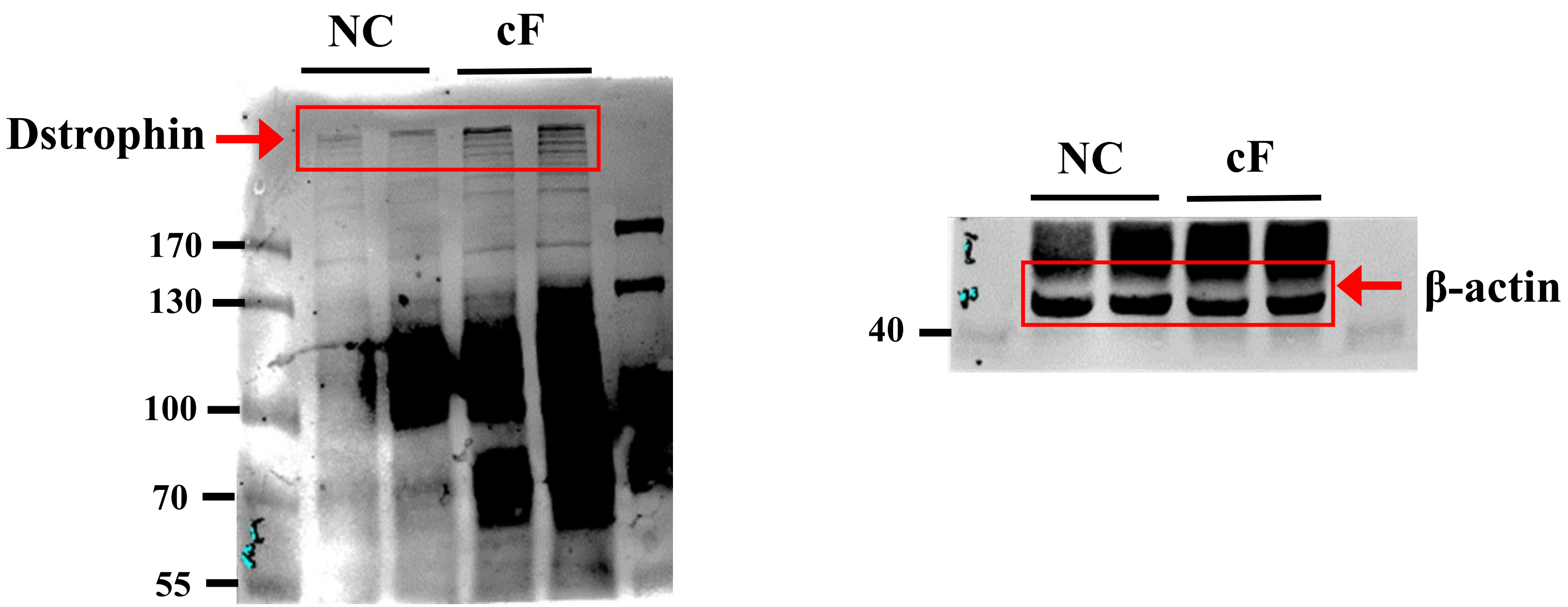

Figure6

C

Total levels of Nav1.5

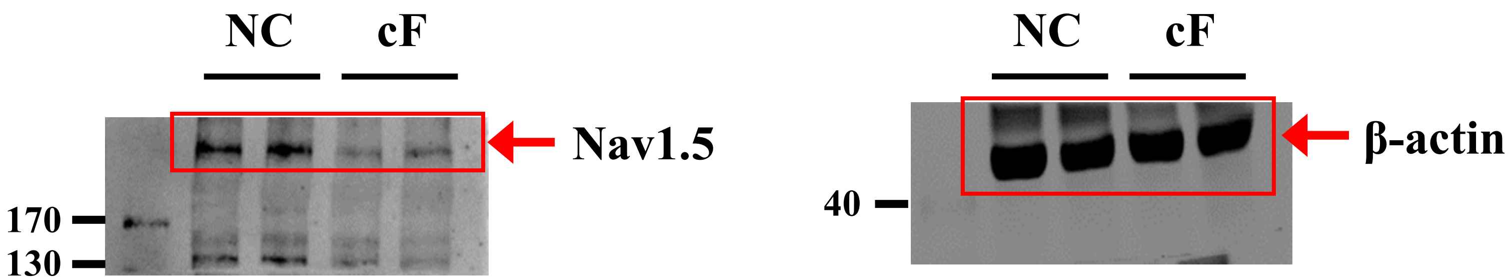

Figure6

C

Membrane levels of Nav1.5

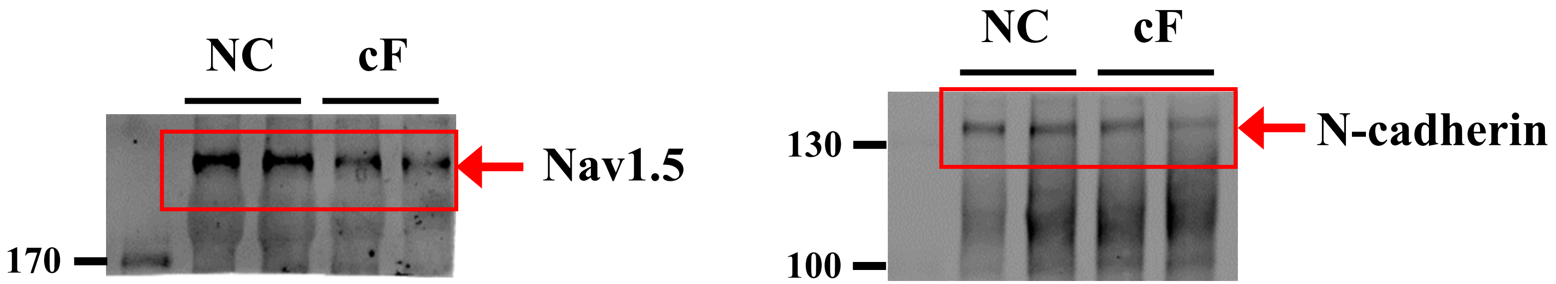

Figure6

C

Intracellular levels of Nav1.5

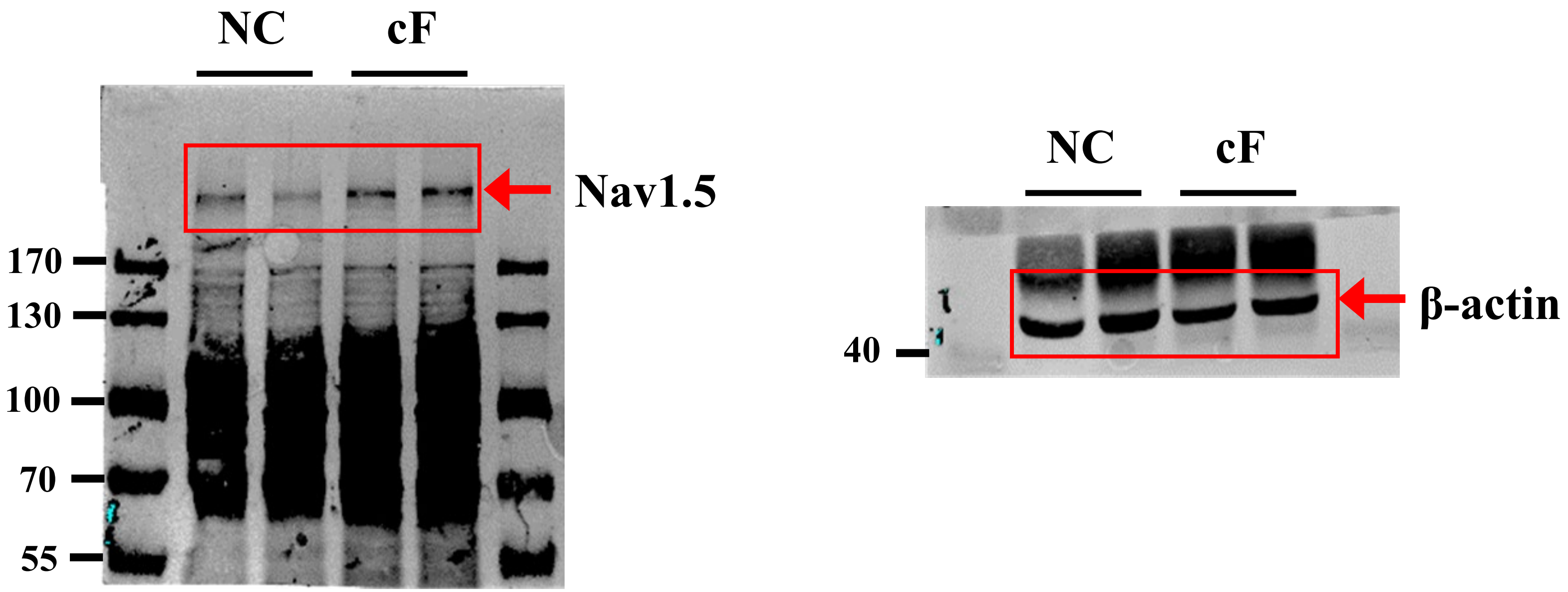

Supplement: Figure 6—source data 4. [file elife-89690-fig6-data4.pdf]
