# Supplementary material for: Binding of LncDACH1 to dystrophin impairs the membrane trafficking of Nav1.5 protein and increases ventricular arrhythmia susceptibility
Source: eLife. 2025 Jan 7;12:RP89690. doi: 10.7554/eLife.89690 (PMC11706603; doi:10.7554/eLife.89690)

Figure 6-figure supplement 5

A

Total levels of dystrophin

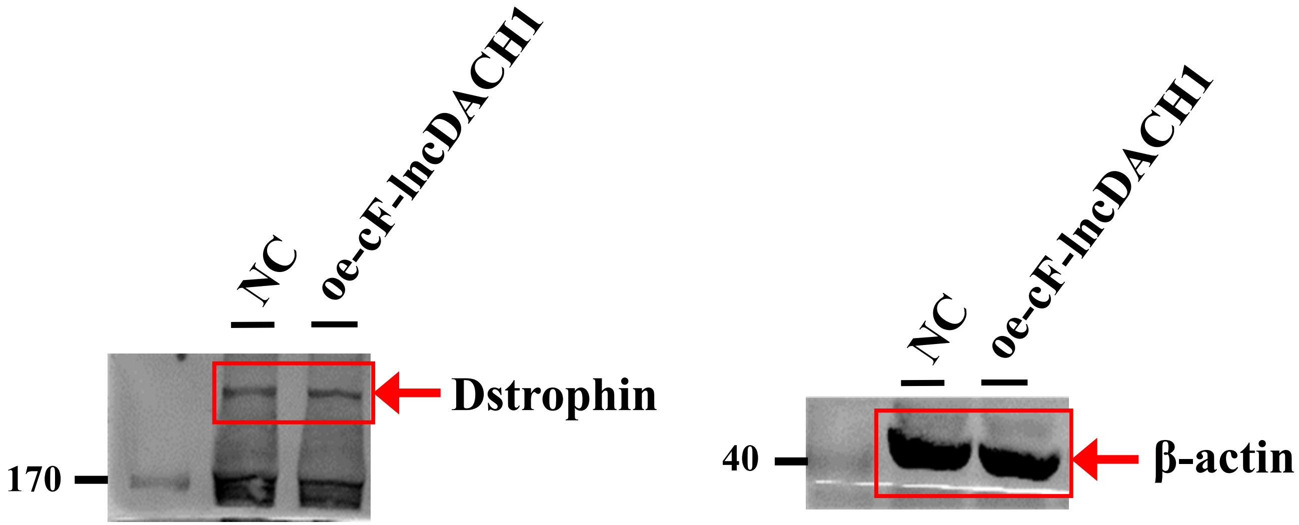

Figure 6-figure supplement 5

A

Intracellular levels of dystrophin

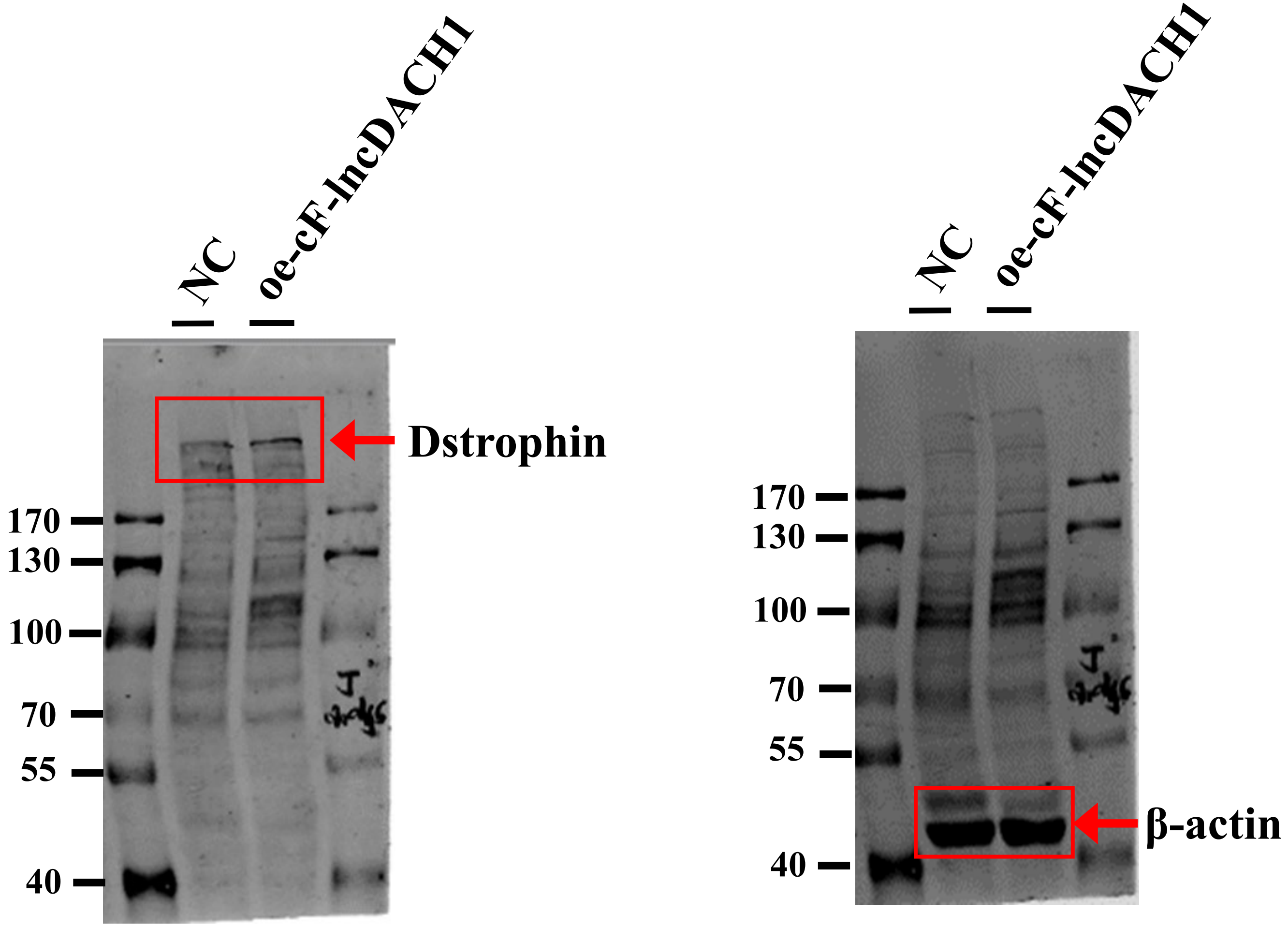

Figure 6-figure supplement 5

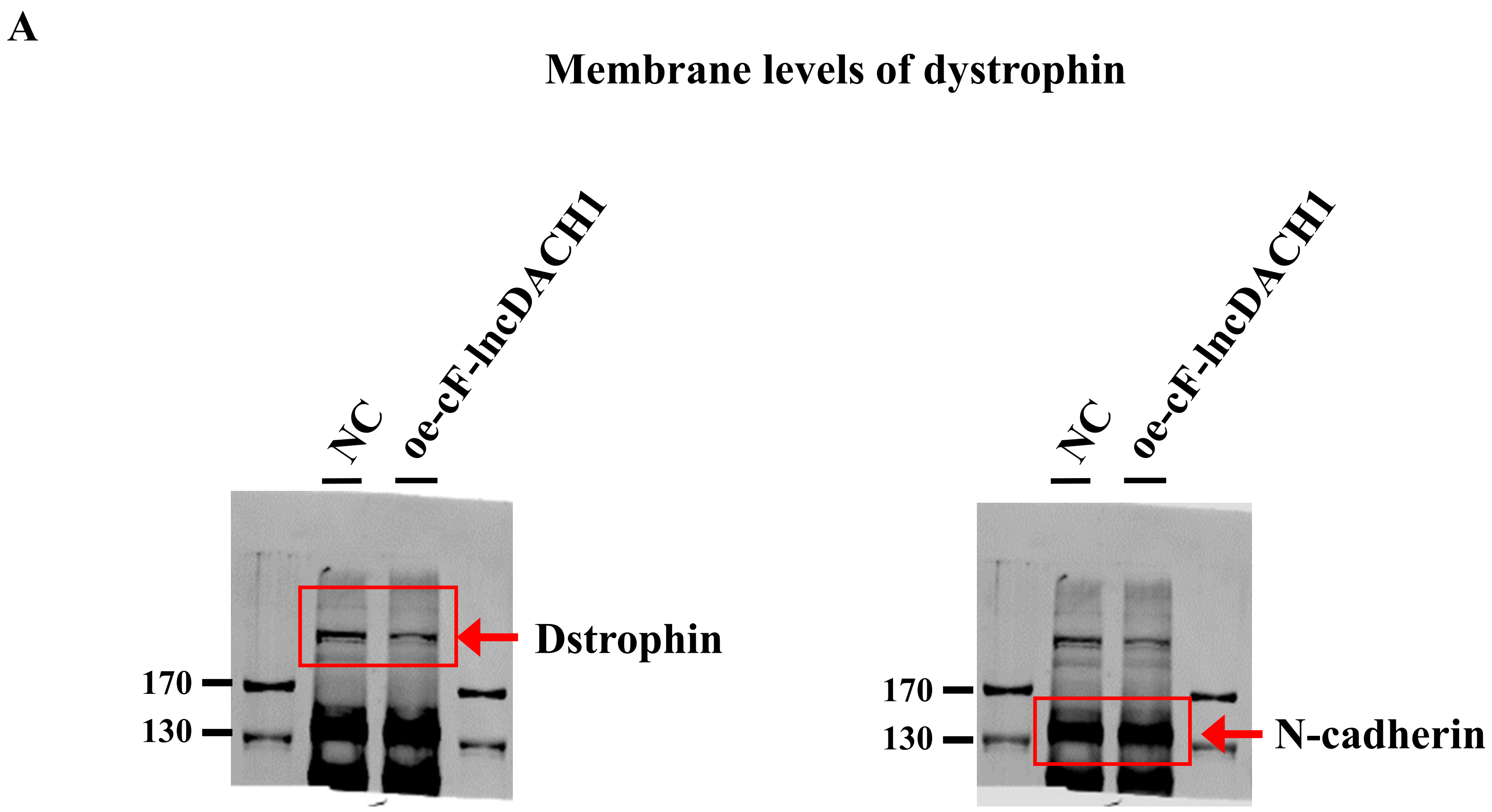

Supplement: Figure 6—figure supplement 5—source data 2. [file elife-89690-fig6-figsupp5-data2.pdf]
