# Supplementary material for: Binding of LncDACH1 to dystrophin impairs the membrane trafficking of Nav1.5 protein and increases ventricular arrhythmia susceptibility
Source: eLife. 2025 Jan 7;12:RP89690. doi: 10.7554/eLife.89690 (PMC11706603; doi:10.7554/eLife.89690)
Supplement: Figure 7—source data 4. [file elife-89690-fig7-data4.pdf]

Figure7

B

Total levels of Nav1.5

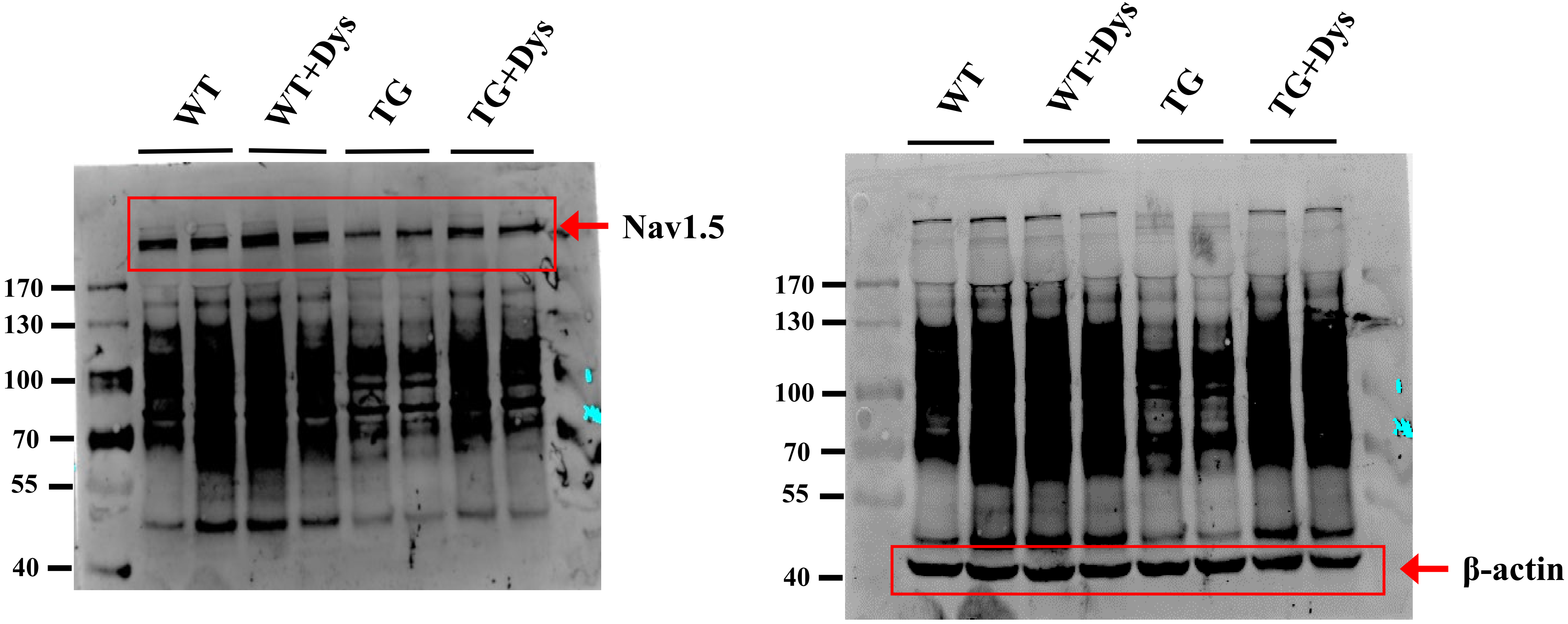

Figure 7

B

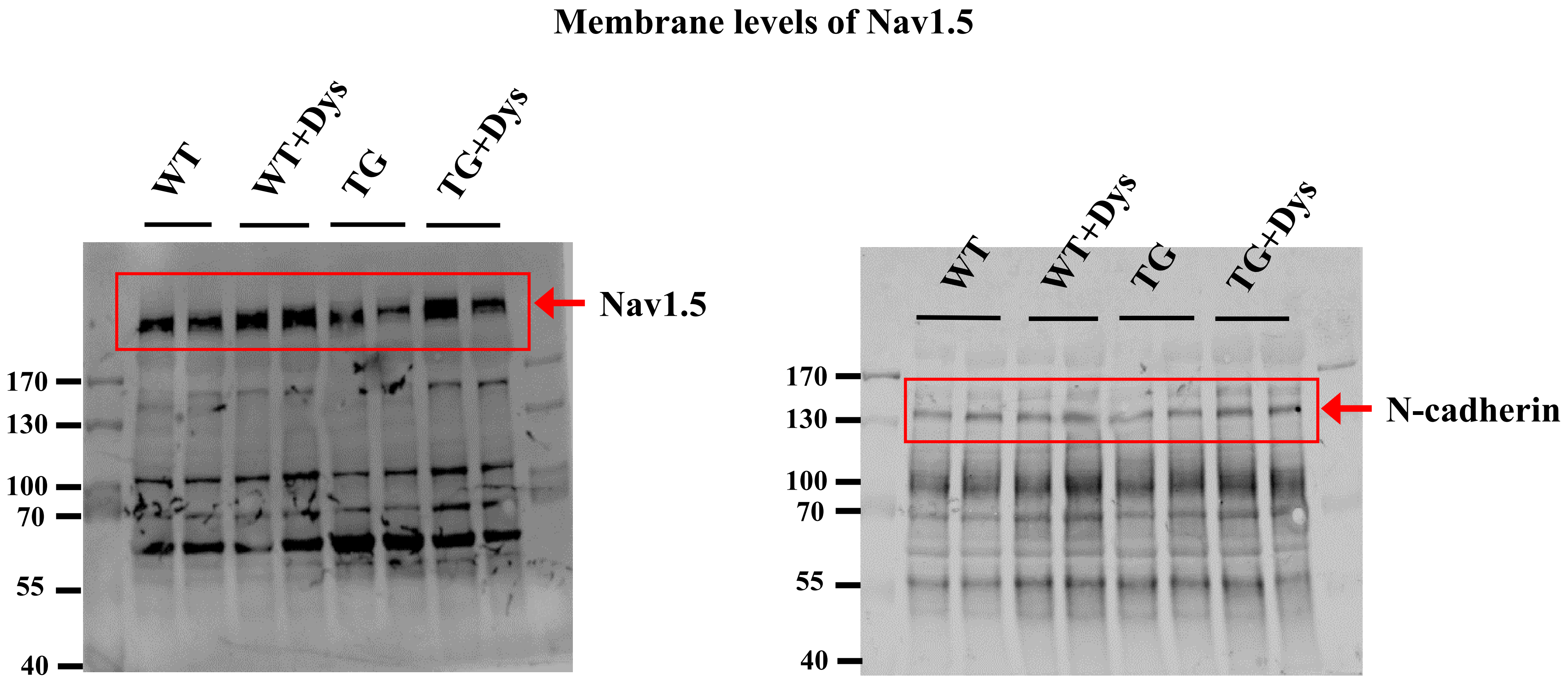

Membrane levels of Nav1.5
WT
WT+Dys
TG
TG+Dys
Nav1.5
170
130
100
70
55
40
WT
WT+Dys
TG
TG+Dys
N-cadherin
170
130
100
70
55
40

Figure7

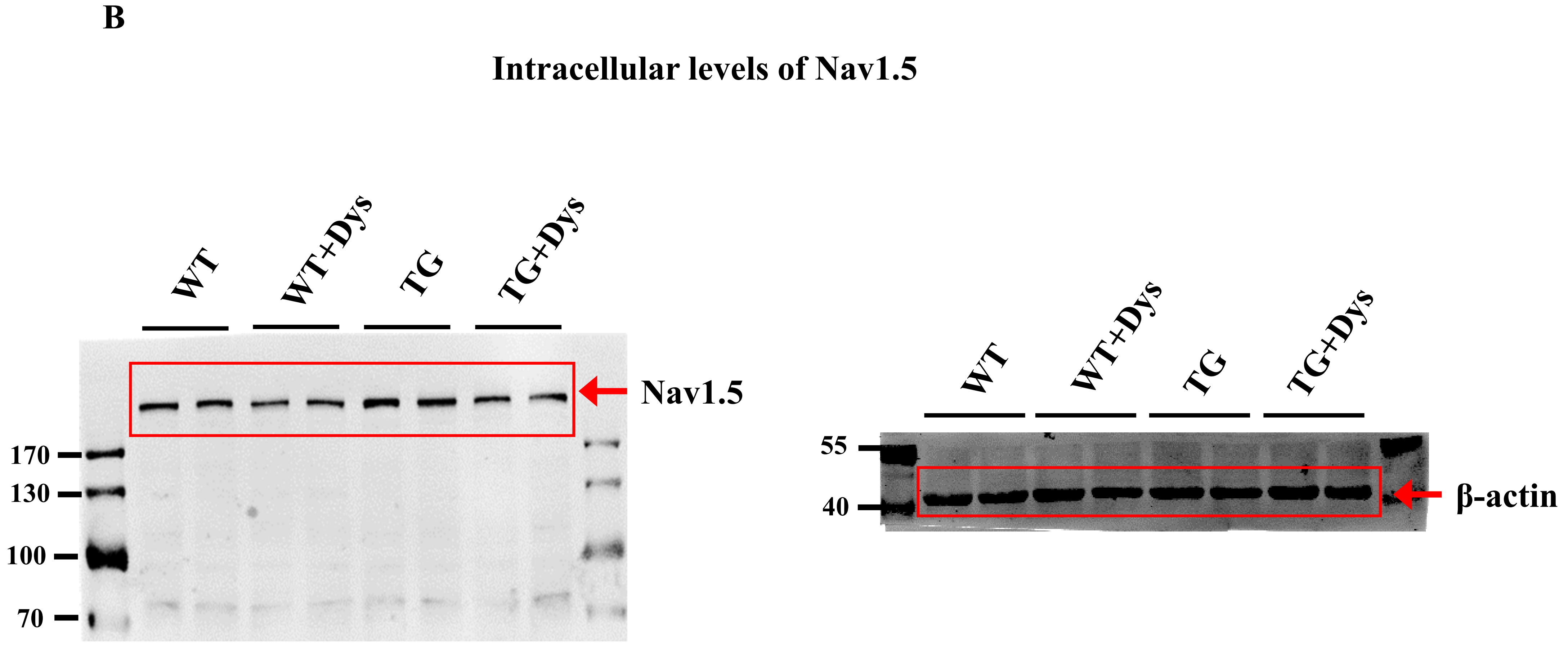
B
Intracellular levels of Nav1.5
WT
WT+Dys
TG
TG+Dys
Nav1.5
170
130
100
70
WT
WT+Dys
TG
TG+Dys
55
40
β-actin
