# Supplementary material for: Binding of LncDACH1 to dystrophin impairs the membrane trafficking of Nav1.5 protein and increases ventricular arrhythmia susceptibility
Source: eLife. 2025 Jan 7;12:RP89690. doi: 10.7554/eLife.89690 (PMC11706603; doi:10.7554/eLife.89690)
Supplement: Supplementary file 2. [file elife-89690-supp2.docx]

**(A)** Alignment of murine lnc*DACH1* and human orthologue


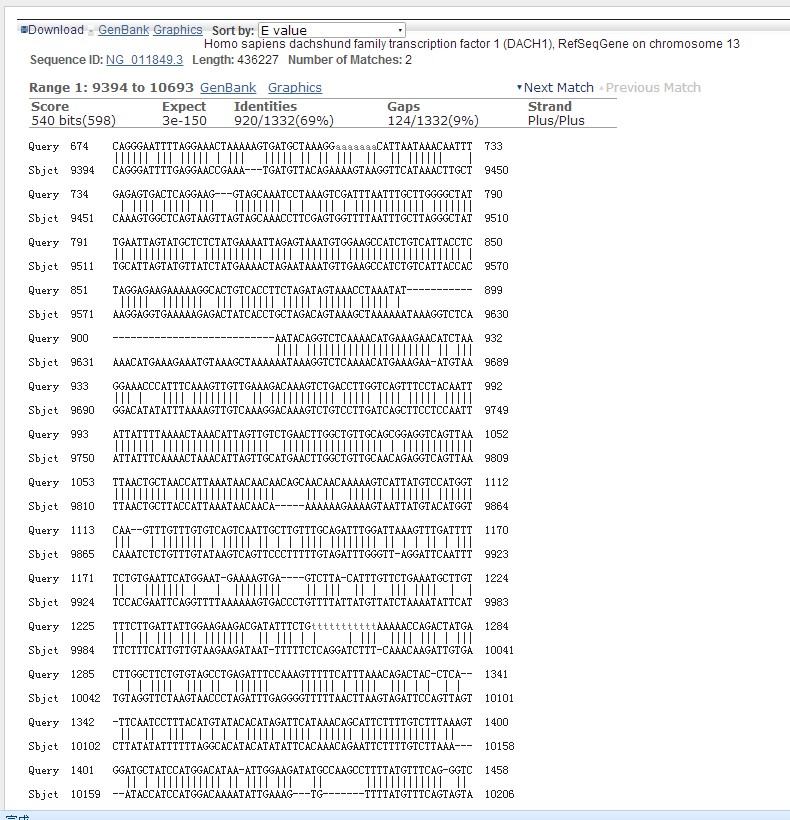


**Human**

**Murine**

**(B)** Alignment of murine lnc*DACH1* and sheep orthologue


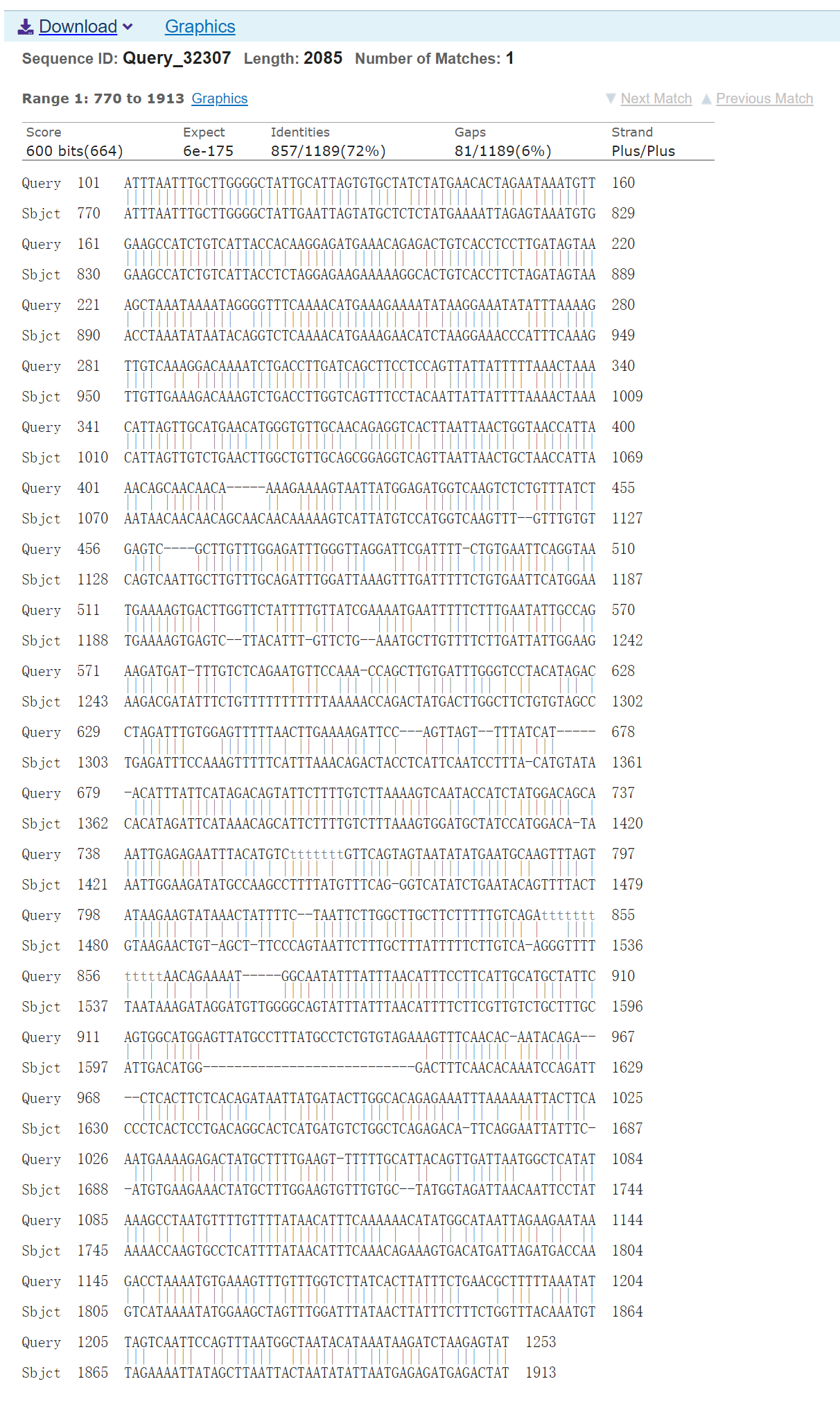


**Sheep**

**Murine**

**(C)** Alignment of murine lnc*DACH1* and Pig orthologue


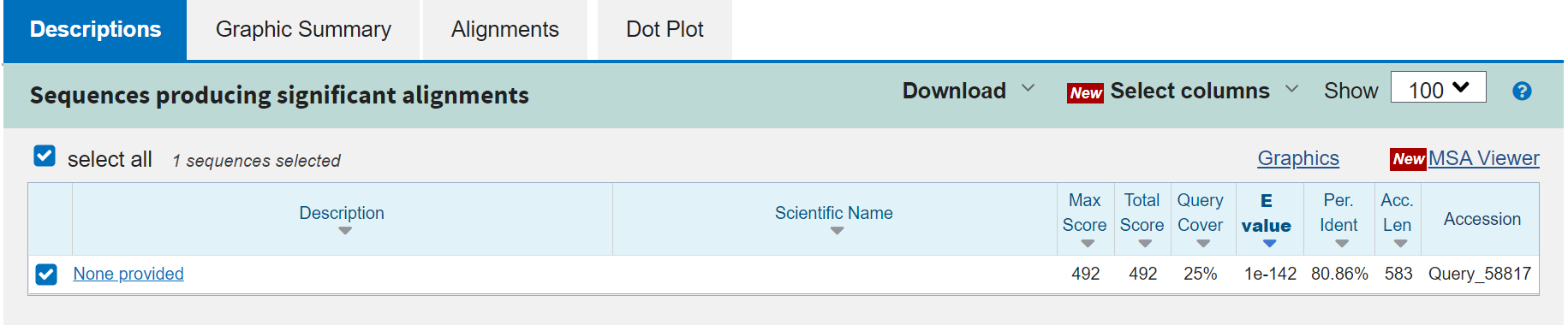


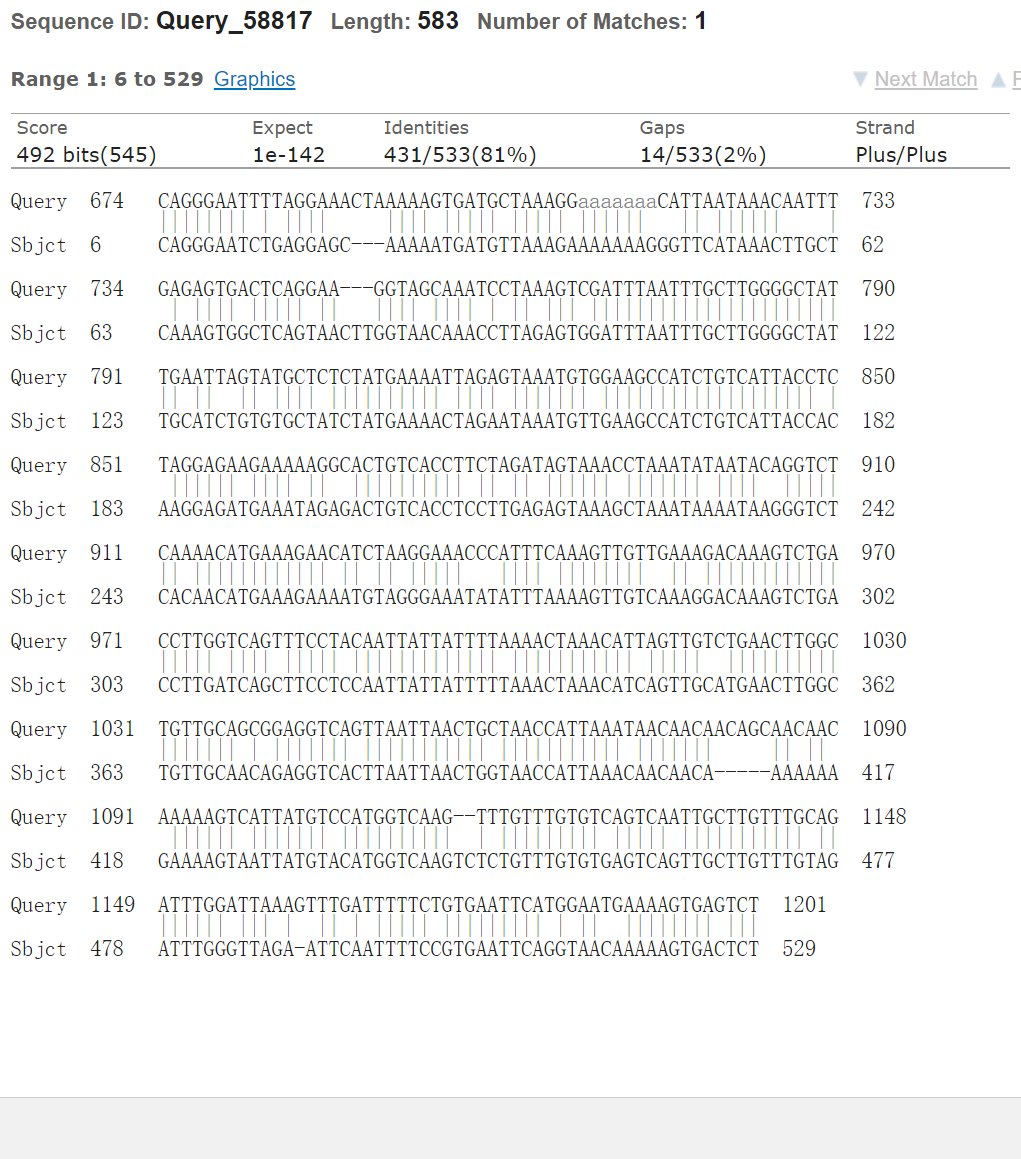


**Pig**

**Murine**

**(D)** Alignment of murine lnc*DACH1* and dog orthologue


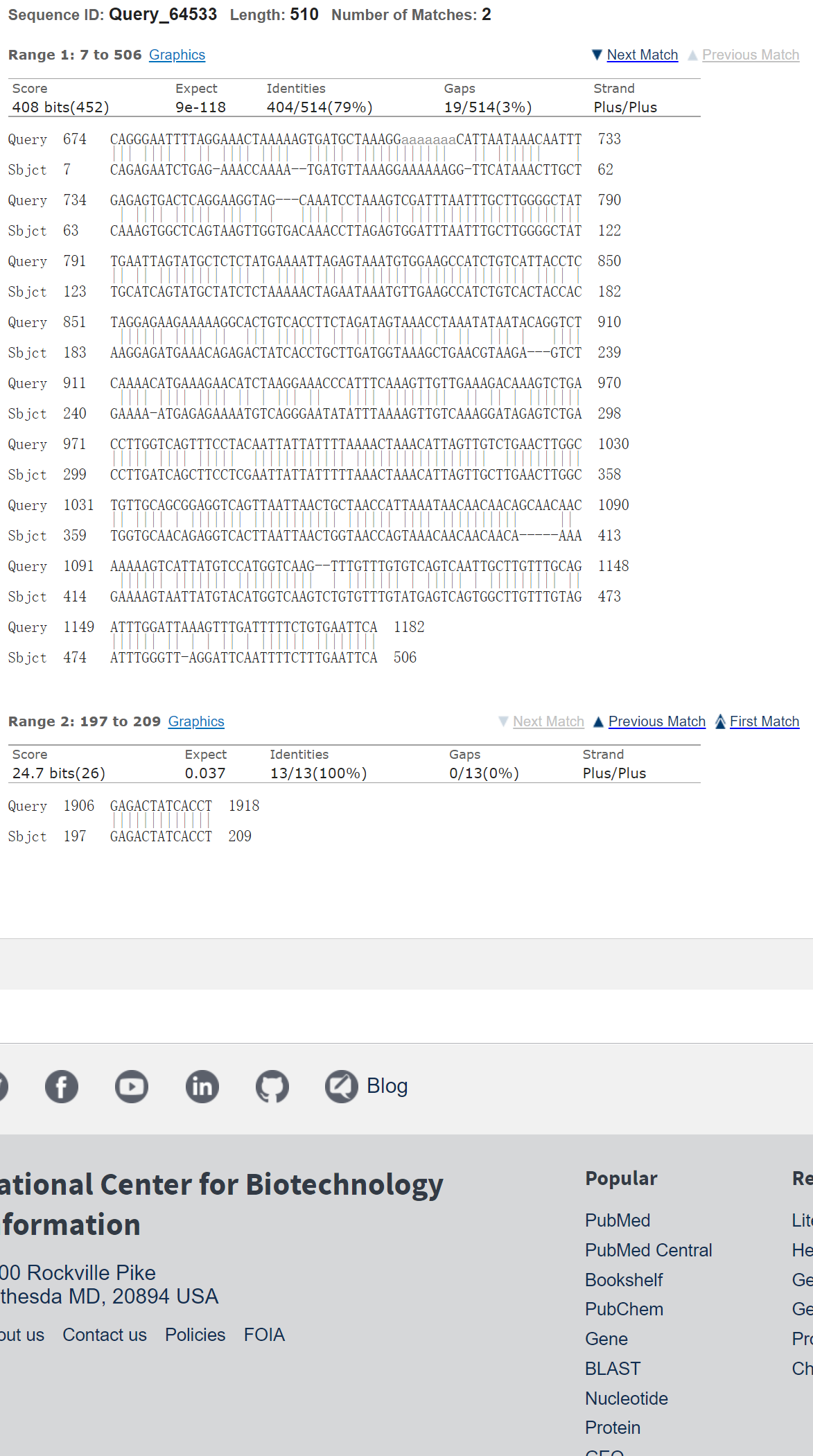


**Dog**

**Murine**

**(E)** Alignment of murine lnc*DACH1* and Rat orthologue


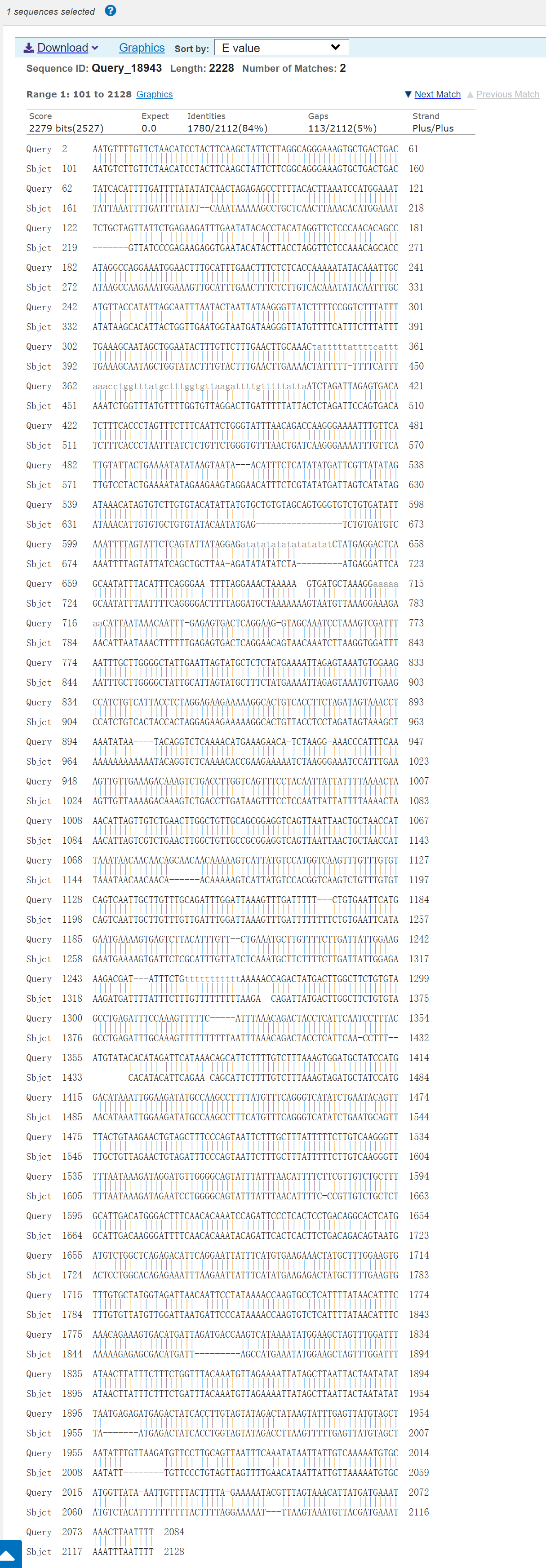


**Rat**

**Murine**
